# Polymer Selection for Hot-Melt Extrusion Coupled to Fused Deposition Modelling in Pharmaceutics

**DOI:** 10.3390/pharmaceutics12090795

**Published:** 2020-08-22

**Authors:** Gabriela G. Pereira, Sara Figueiredo, Ana Isabel Fernandes, João F. Pinto

**Affiliations:** 1 iMed.ULisboa–Research Institute for Medicines, Faculdade de Farmácia, Universidade de Lisboa, Av. Prof. Gama Pinto, 1649-003 Lisboa, Portugal; garrastazugp@farm-id.pt (G.G.P.); sara.figueiredo@ff.ulisboa.pt (S.F.); jfpinto@ff.ul.pt (J.F.P.); 2 CiiEM, Interdisciplinary Research Center Egas Moniz, Instituto Universitário Egas Moniz, Quinta da Granja, Monte de Caparica, 2829-511 Caparica, Portugal

**Keywords:** extrudability, fused deposition modelling (FDM), hot-melt extrusion (HME), polymers, printability, three-dimensional printing

## Abstract

Three-dimensional (3D) printing offers the greatest potential to revolutionize the future of pharmaceutical manufacturing by overcoming challenges of conventional pharmaceutical operations and focusing design and production of dosage forms on the patient’s needs. Of the many technologies available, fusion deposition modelling (FDM) is considered of the lowest cost and higher reproducibility and accessibility, offering clear advantages in drug delivery. FDM requires in-house production of filaments of drug-containing thermoplastic polymers by hot-melt extrusion (HME), and the prospect of connecting the two technologies has been under investigation. The ability to integrate HME and FDM and predict and tailor the filaments’ properties will extend the range of printable polymers/formulations. Hence, this work revises the properties of the most common pharmaceutical-grade polymers used and their effect on extrudability, printability, and printing outcome, providing suitable processing windows for different raw materials. As a result, formulation selection will be more straightforward (considering the characteristics of drug and desired dosage form or release profile) and the processes setup will be more expedite (avoiding or mitigating typical processing issues), thus guaranteeing the success of both HME and FDM. Relevant techniques used to characterize filaments and 3D-printed dosage forms as an essential component for the evaluation of the quality output are also presented.

## Table of Contents

1. Introduction
2. Hot-Melt Extrusion
3. Fused Deposition Modelling
4. Polymers Used in Hot-Melt Extrusion and Fused Deposition Modelling FDM
  4.1. Polyvinyl Alcohol
  4.2. Polyvinylpyrrolidone
  4.3. Cellulose-Derived Polymers
    4.3.1. Ethylcellulose
    4.3.2. Hydroxypropylcellulose
    4.3.3. Hydroxypropylmethylcellulose
    4.3.4. Hydroxypropylmethylcellulose Acetate Succinate
  4.4. Acrylates
    4.4.1. Eudragit^®^ E PO
    4.4.2. Eudragit^®^ RL
    4.4.3. Eudragit^®^ RS
    4.4.4. Eudragit^®^ L
  4.5. Other Polymers
  4.6. Commercial Polymer Blends
5. Characterization of Filaments and 3D Dosage Forms Produced by HME-Coupled with FDM
  5.1. Rheologic Properties
  5.2. Mechanical Properties
  5.3. Thermal Properties and Other Characterization at Molecular Level Techniques
  5.4. In Vitro and In Vivo Characterization
  5.5. Other Properties
6. Final Remarks

## 1. Introduction

Three-dimensional (3D) printing, also known as additive manufacturing, has recently been attracting the attention of the scientific community in many different areas, including pharmaceutics. This technology, which encompasses a range of 3D-printing techniques, presents many advantages such as (a) the possibility to produce dosage forms on demand, with a specific and precise dose [1]; (b) the incorporation of several drugs into a single dosage form [2]; (c) the modulation of drug release profile by tailoring shape and density [3] of dosage forms; (d) the production of complex multi-doses and release profiles by printing several drugs and using barrier coatings [4] or loading with (micro/nano)particles [5]; and (e) high reproducibility [6].

In fact, the opportunity to personalize therapy according to patient’s needs, re-centering medicines’ design on the individual, is actually one of the greatest advantages of 3D-printed medicines. Groups with specific therapeutic needs, such as pediatrics and geriatrics, could benefit from individualization, ensuring accurate dosing of drugs requiring continuous dose adjustment (e.g., theophylline and prednisolone) [7]. Customization to match physiological, pathological, biochemical, or genetical idiosyncrasies or cater for preferences or lifestyle needs are also paramount. Besides, the possibility of combining multiple drugs in one dosage unit, each presenting a tailored release profile, can improve patient compliance, treatment effectivity, and cost-effectiveness [8]. Such approach has been exploited in recent years, and dosage forms containing more than one active pharmaceutical ingredient (API), designated as multi- or polypills, have been developed [8,9,10]. Furthermore, the COVID-19 pandemic has unveiled the need for decentralized production of medicines, closer to the patient, to face supply chains’ disruption and drug shortages. In the future, 3D printing of pharmaceuticals may fill this gap.

**Figure 1:**
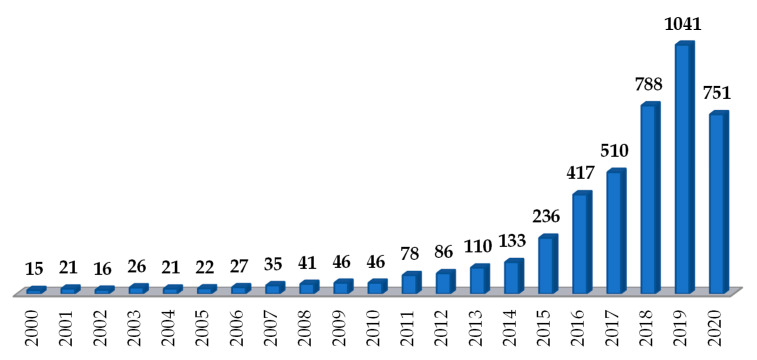
Number of publications relating to 3D printing of medicines over the last 20 years (ScienceDirect^®^).

Three-dimensional printing has shown promise, in particular for the development of oral solid dosage forms incorporating synthetic drugs. The US Food and Drug Administration approval in 2015 of the first 3D-printed medicine—SPRITAM, an orally disintegrating tablet of levetiracetam manufactured by the ZipDose Technology (Aprecia Pharmaceuticals, Langhorne, PA, USA) [11]—was a landmark. Since then, research in the field has boosted, and in the last 10 years, the number of related publications has continuously grown. Figure 1 shows the number of research and review papers, published in the last 20 years, retrieved from ScienceDirect^®^ (https://www.sciencedirect.com/, accessed on 1 June 2020), using the term “3D printing and medicines”. It is recognizable that the number of publications related to the 3D printing of medicines more than doubled in the last five years, reflecting the interest and relevance of the subject at present.

There are many additive manufacturing technologies (mainly based on powder agglomeration, photo-polymerization, or extrusion) and printers available, each of them with their particularities, advantages, and disadvantages. At present, there is not a technology that fits every need; it is up to the pharmacist to choose the best fit, in view of the safety, efficacy, and quality requirements of the pharmaceutical dosage form to print [12].

One of the most widespread technologies for pharmaceutical applications is the fused deposition modelling (FDM), invented by Scott Crump in 1980 [13] and detailed further ahead. In short, the process starts by digitally designing the desired dosage form using computer-aided design (CAD) software; these designs are then converted into stl files, which instruct and control the printer. The FDM printer is fed with a drug-containing thermoplastic polymeric filament, usually obtained by hot-melt extrusion (HME), and performs an extrusion of the molten materials, which are continuously deposited, layer by layer, to build the 3D structure [14,15,16]. The polymeric raw materials most used in the pharmaceutical industry for this purpose—polylactic acid (PLA) [17], polyvinyl alcohol (PVA) [3], hydroxypropyl cellulose (HPC) [13], acrylates [5,18], etc. [19,20]—are discussed in the following sections.

## 2. Hot-Melt Extrusion

Hot-melt extrusion (HME) has become one of the most common methods for the preparation of drug solid dispersions with various polymers or/and lipid matrices. It is currently used for drug solubility enhancement but also for time-controlled, extended, and targeted drug delivery [21]. Coupling of HME with 3D printing to prepare pharmaceutical dosage forms has recently started to be explored [22,23,24].

A major obstacle for FDM 3D printing use in pharmaceutical manufacturing is that most commercial filaments available at present are unsuitable for pharmaceutical applications [22]. For that reason, prior in-house preparation of the filaments is required. HME is the most attractive method to produce filaments for pharmaceutical applications using existing pharmaceutical-grade polymers [25,26] and is regarded as an excellent alternative to other techniques available [22,23,24]. Although most polymers are amenable to use in HME to prepare polymeric filaments [22], the choice must consider the desired application, as well as their thermal and mechanical properties, as discussed below. Drug loading into the filament can be achieved by incorporation into the powder mixture before the extrusion process or by making the drug diffuse passively into the filament, soaking an extruded filament in a suitable drug-containing solution [3,27]. The main drawback of the latter approach is the limited drug loading (<2%) achieved, though avoiding degradation of thermolabile drugs. On the other hand, since the HME of powders containing the drug allows the incorporation of high amounts of drug, this technique ensures greater dose flexibility [28]. Moreover, it may be adapted to produce standard formulae appropriate for the incorporation of several active ingredients, thus avoiding the need for altering the whole composition. Therefore, different drugs may be incorporated into filaments with essentially identical excipients [29], thus expediting the formulation process.

Drug-loaded filaments produced by HME are suitable for 3D printing, provided some requirements are met. During the feeding of the filament into the FDM 3D-printer head, the filament will undergo tensile and compressive forces caused by the extruding gear, as well as heating for melting during the printing process. Therefore, to achieve a good printing quality, the filaments are required to withstand both the mechanical and thermal stresses to which they are subjected in the FDM print head [30]. However, most polymeric excipients, traditionally used in pharmaceutics, do not present suitable thermal and mechanical properties, precluding 3D printing [31]. With the adequate selection of formulation and processing conditions, HME can produce good quality thermoplastic filaments, to feed FDM 3D printers.

## 3. Fused Deposition Modelling

FDM is considered the most reproducible, inexpensive, and accessible 3D-printing technology [32], as compared to ink jetting, selective laser sintering, stereolithography, or powder bed. Specifically considering drug delivery purposes, this technique provides rapid prototyping and accurate and versatile incorporation of variable drug concentration gradients within the polymer matrix [32].

Moreover, FDM can lead to innovative drug delivery strategies, which may overcome the limitations and drawbacks of traditional dosage forms. The possibility of manufacturing highly accurate patient-tailored tablets [24,33] or capsules [34,35], presenting varied geometries [3,36], either with immediate or controlled drug release features [2,13], has proven the huge potential of 3D-printing technologies based on FDM to fabricate drug delivery systems and expanded its use in pharmaceutics. In fact, drug content and release profile of the incorporated drug can be adjusted due to the limitless possibilities of printing a diverse range of sizes and shapes. Although several factors can influence the final specimen quality (e.g., infill density, extrusion speed, layer height, and nozzle temperature), FDM holds great potential and usefulness, provided these are successfully controlled [3,27,37,38].

FDM is based on the extrusion of a filament through a nozzle. The filaments used are generally manufactured in a circular die extruder [24,39,40]. FDM printers use two kinds of materials—a modelling material, which constitutes the finished object, and a support material, which acts as a scaffolding to support the object during printing, when complex geometries are needed. Materials are molten and extruded and immediately deposited layer by layer on a building plate, or platform, moving in the xy plane. This thin layer of polymer cools and hardens, immediately binding to the layer beneath. Once a layer is completed, the plate, which presents a z-direction mobility, is lowered (usually by about 300 µm) to make room for the next layer of polymer [3,41]. The process repeats itself until the specimen is completed.

One of the major drawbacks of FDM is that it requires previous in-house preparation of filaments, usually by HME, as previously described. The quality of FDM 3D-printed products is heavily dependent on the characteristics of the filaments (e.g., melting temperature, melt viscosity, rate of polymer solidification, thickness/diameter, surface roughness, and mechanical properties such as plasticity, rigidity, or brittleness). Heat transfer characteristics and rheology of melted polymers are especially significant criteria influencing the selection of a suitable material for the intended purpose, since FDM is also a thermo-based technique [32]. Due to their relatively low melting temperature, thermoplastic polymers prevail in FDM applications [41,42]. As FDM printing technology establishes itself in pharmacy (either in biomedical applications or drug delivery systems), the polymer industry is catering for its particular requirements, and novel polymers are under development. Until now, a restricted number of commercial polymers have been investigated for oral drug delivery system purposes, although many polymers have already been used in the manufacture of implants and scaffolds [25,43].

FDM critical process parameters are key variables affecting the production process and may be related to the machine, the operation, and the material [44]. Machine-specific parameters relate to the printer itself, so choice of make and model are of importance; operation-specific parameters derive from the processing conditions during the printing process; material-specific parameters relate to the physiochemical properties of the filament to print. Printers allow control over operation-specific parameters, such as printing speed and temperature. However, most pharmaceutical polymers do not present the required rheology upon melting and print feeding ability, usually preventing their use for FDM. Adequate set-up of processing parameters (e.g., printing resolution, infill density, layer height and extrusion speed, nozzle diameter and temperature, and plate temperature) is required to achieve suitable printing [24]. Polymers must be heated above polymer glass transition temperature to soften or melt, and thus, control of nozzle temperature is paramount. Due to the fleeting time of the material in the nozzle and the low shear of the extrusion, filaments must withstand higher temperatures than those used in HME. Extrusion temperature difference between HME and FDM can be above 100 °C [25], depending on the material. Molten material is laid up, layer by layer, in the printing plate. Construction of the specimen requires layer bonding, a process that can be affected by rapid cooling. Therefore, the printing plate should be slightly heated and thermo-stated to permit appropriate consolidation of the 3D structure by gradual cooling [26]. The infill of the 3D structure is also of importance. At higher infill, a slower release of the API was observed, even though a relation between porosity and drug release rates could not be established [27]. The 3D-printing infill can assume very small values for hollow structures or attain 100% to manufacture compact specimens, with direct impact on drug content and release profile [45]. Control of printing variables presents itself as a simple strategy to fulfil the therapeutic advantages of 3D-printed drug products.

**Figure 2:**
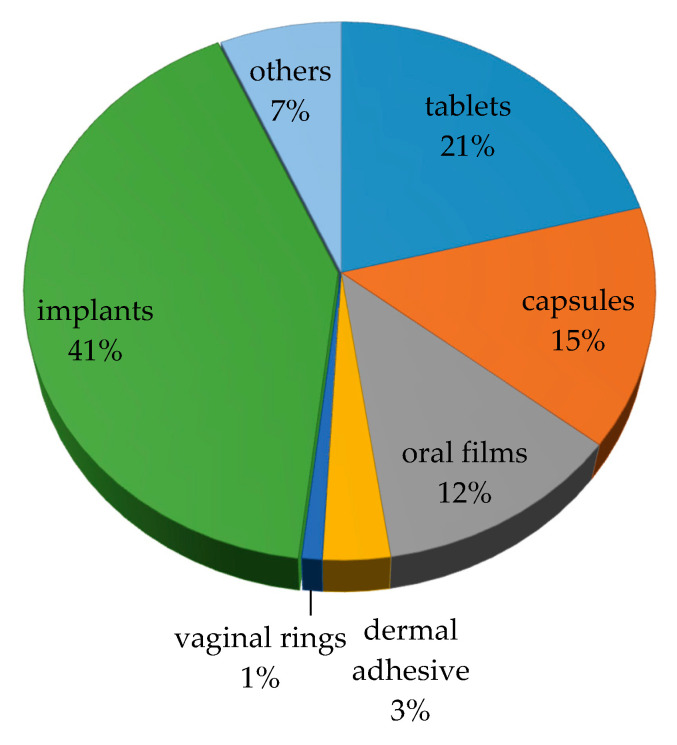
Pharmaceutical dosage forms printed by fused deposition modelling (FDM), as published between 2009 and 2019 (ScienceDirect^®^) (n = 613 publications).

A comprehensive variety of different drug delivery devices have been manufactured by FDM 3D printing, as highlighted by the extensive number of recent publications on the subject. A literature search (https://www.sciencedirect.com/, accessed on 31 December 2019), combining the terms, “FDM” and “Drugs” from 2009 to 2019 returned 613 original research papers and reviews. The scope of these works gives an idea of the technology’s potential and the diversity of dosage forms produced (Figure 2). As expected, the majority of studies explored the development of oral dosage forms, with tablets accounting for the largest share (21%), followed by capsules (15%). Oral formulations in general account for about 50% of the drug products produced by FDM, although implantable drug delivery systems are the most representative group (41%).

Regarding drugs, 3D printing of oral dosage forms has been attempted mainly in BCS class II or IV drugs as a mean to increase solubility and bioavailability (mainly achieved by the previous HME), but also due to clinical constraints presented by the drugs. In fact, 3D printing is regarded as an asset to help drugs meet clinical needs, either by (a) repeated dose adjustment depending on body mass or age (e.g., theophylline in pediatrics) or in case of narrow therapeutic margin drugs (e.g., warfarin used for thromboembolic events prevention); (b) ameliorating organoleptic properties by taste masking and production of dosage forms suited to a particular age group (e.g., indomethacin), such as children; or (c) managing of polymedication or complex therapeutic regimens, drug incompatibility, need for different release profiles of the same drug, by using multiple drugs in the same unit (e.g., metformin and glimepiride). These are examples of the many approaches allowed by the 3D printing of medicines, which will be detailed later in this text. Table 1 summarizes the drugs that have already been incorporated in 3D-printed dose forms, therapeutic class, and physico-chemical characteristics relevant for FDM 3D printing.

However, as an emergent technology, the limitations of FDM need to be identified and addressed. First of all, FDM 3D printing may not be the ideal solution when large-scale production is needed, since tablet machines can be much faster than a printer [40]. However, it has the potential to solve a current therapeutic gap regarding the need of personalized therapy, playing a complementary or alternative role to the conventional industrial production [33,46]. Second, the methods of drug incorporation preceding FDM are still a concern because they resort to heat. Even though HME offers high and adjustable drug loading, drug candidates are limited to thermo-stable substances. Equally, the printing process requires high temperatures to melt the filament during extrusion when building the object, which may cause physical instability and degradation issues. Thermo-sensitive drugs are thus unsuitable for FDM-based printing, restricting its biomedical and pharmaceutical applications and clearly requiring investment in the development of FDM-specific polymers with lower melting temperatures. Third, the thermoplastic properties of the polymeric filaments can be changed when an additive is incorporated into the matrix, which may have an impact on properties affecting extrudability and printability, such as viscosity and flexibility. Finally, support structures, which need to be constructed when the object has large base area to prevent warping (by increasing the adhesion between the specimen and the build platform) or complex geometry, contribute to material wastage. Additionally, the inner scaffold required to sustain hollow objects is difficult to remove in scaled-up procedures [32].

**Figure 3:**
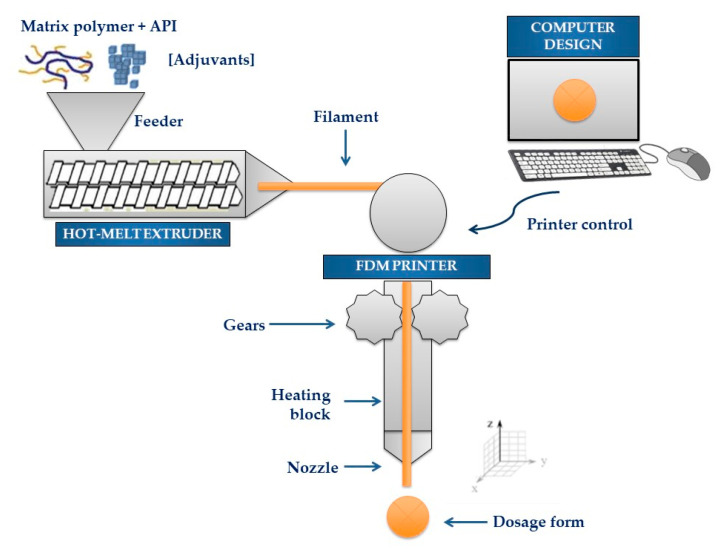
Schematic representation of integrated hot-melt extrusion and fused deposition modelling for pharmaceutical applications.

HME and FDM 3D-printing technologies have shown many advantages when sequentially used in the manufacture of pharmaceutical dosage form and medical devices, streamlining the complex processes of conventional manufacturing methods. Figure 3 depicts the entire flow of 3D printing of a dosage form, starting with the digital design and the selection of raw materials (polymer, drug, and excipients, if needed), and the HME of the powder mixture to produce filaments that, upon cooling, will feed the FDM printer to produce the dosage form according to the computer’s instruction.

Although apparently simple, coupling the two technologies is not without practical difficulties related to the requirements, which filaments must meet, for extrudability and printability. In other words, a polymer suitable for HME may not be per se amenable to FDM. For instance, the temperature needed for printing is generally higher than that required for the extrusion of filaments during HME. Otherwise, problems of nozzle clogging upon increase in the melt viscosity are observed, which modulation of the FDM processing temperature can reduce. Besides, the temperature of the material flowing out of the printing chamber should be able to compensate for the sudden cooling, which occurs when exiting the nozzles, to guarantee material adherence to the layer beneath in order to build the object on the platform [23]. In order to achieve a more effective and efficient manufacturing process, a combination of these two technologies into a single continuous process has been attempted for in-house production of customized dosage forms for immediate consumption [75]. Further details on formulation strategies and processing conditions used, as well as on equipment employed, are given below.

This review contextualizes the polymers currently employed in HME coupled to FDM 3D printing, as matrix formers, aiming to expedite the process set-up and reducing the iterations in the selection of raw materials to meet the extrusion and printing requirements while fulfilling the quality standards of dosage forms.

## 4. Polymers Used in Hot-Melt Extrusion and Fused Deposition Modelling

Polymers are macromolecules composed of repeating units, or monomers, which are either naturally occurring, semi-synthetically modified, or synthetically manufactured [76]. Due to their unique properties they have been used in the pharmaceutical industry as binders, fillers, lubricants, and solubility enhancers in solid dosage forms production, as well as emulsifying, suspending, or stabilizing agents in the manufacture of liquids and semisolids. The development of polymers tailored to meet specific pharmaceutical needs (e.g., flowability or compressibility) has expanded their use as pharmaceutical excipients. More recently, polymers have been used to modulate drug release by developing modified release systems (e.g., sustained, delayed and targeted release) [77]. Polymers are, in fact, the basis of modern, advanced drug delivery systems.

Thermoplastic, or thermosoftening, polymers in the form of filaments wound on a coil [22], are the FDM raw material. Thermoplastic polymers are composed of long linear chains, held together with weak attraction forces, that, when subjected to high temperatures, soften or melt and solidify upon cooling, assuming defined forms at room temperature. Thermoplastic polymers are composed of two phases depending on the degree of intermolecular interactions—the amorphous structure is responsible for the flow properties of the materials, and the crystalline structure is responsible for the impact resistance [78]. The extrusion pressure generated in FDM 3D printing is responsible for the heat molten/softened material’s flow through the nozzle.

This section reviews the most common polymers used in HME-coupled FDM as drug carriers, the trade names and characteristics of which are presented in Table 2. Glass transition temperature (Tg) values vary with the molecular weight of the same polymer, the method of determination, and the rate of heating or cooling the sample [78]. Polymer manufacturers, due to industrial property issues, sometimes do not fully disclose the properties of polymers, and collection of information is time-consuming and sometimes hampered by the existence of several manufacturers reporting slightly different values for the same property. For such reasons, and because polymers are polydisperse, it is common to find discrepancies in the values reported in the literature. Although the addition of the drug or adjuvants will most certainly change the thermal properties of the polymer, indicative Tg and Tm values are given as a starting point to equate the necessity for plasticization. In fact, the knowledge of the thermal properties of polymers is paramount for processing. HME, for instance, should be run at temperatures 20–40 °C above Tg [79]. Likewise, small differences between Tg and the degradation temperature of the polymer constitute a problem for HME or FDM usage and must be borne in mind from the start.

Traditionally, PVA, PLA, and polyvinylpirrolidone (PVP) are the most commonly used pharmaceutical grade polymers for FDM. More recently, other polymers have been added for filament preparation, such as cellulose ethers (hydroxypropyl cellulose (HPC); hydroxypropyl methylcellulose (HPMC); ethylcellulose (EC); hydroxypropyl methylcellulose acetate succinate (HPMCAS)) and derivatives of acrylates (e.g., Eudragit^®^).

The association of other components, such as plasticizers or fillers, is often required to enable HME and 3D printing with common polymers by directly influencing their properties. These additives improve the suitability of filaments for FDM, either by optimizing the rheological and physical properties of the intermediate products (e.g., flowability of melt and flexibility of filaments) and by broadening the range of substances that can be used in this technology (e.g., drugs susceptible to thermal degradation) [80,81]. Table 3 details the formulations attempted to produce 3D-printed dosage forms by coupling HME and FDM; the qualitative and quantitative information on the main matrix polymer and additives used are given. Trade names and manufacturers of the polymers are provided to simplify polymer choice by the less experienced scientist and facilitate incursion in this investigation area.

Polymers are typically used in the range of 35% to 95% (100% for controls); the remaining fraction relates to drug(s) and processing adjuvants (Table 3).

Plasticizers, the most extensively used adjuvants, are typically low-molecular-weight compounds, which are often added to the polymeric formulations in order to enhance their processing conditions. On the one hand, these molecules enable the reduction of the polymer Tg, lowering the extrusion temperature and allowing gentler melting conditions, with clear advantages for drugs with thermal susceptibility. In addition, the use of plasticizers improves the intrinsic properties of polymeric materials since these adjuvants decrease the viscosity of the formulation and increase the ductility of the filaments. Both features are particularly relevant for the feeding stage of the filament in FDM, respectively, by increasing flowability of the material and reducing brittleness of filaments [80]. In turn, over-plasticization of filaments can cause feeding defects since the permanent deformation of the over-plasticized filaments along the printer head precludes the subsequent fusion and deposition of the material. As presented in the next section, the flexibility of filaments is an essential feature, since it minimizes the possibility of rupture inside the printing head. However, the filaments must not be so pliable that they are permanently deformed; on the contrary, they must retain their original shape when force is removed.

Since the level of plasticization is therefore critical, reference amounts (% *w/w*) of plasticizers, used in several works, are also given in Table 3. Amongst the plasticizers used in pharmaceutical blends for FDM printing are various grades of polyethylene derivatives (polyethylene glycol—PEG and polyethylene oxide—PEO), Tween^®^ 80, triethyl citrate (TEC), triacetin, and glycerol [42]. Three-dimensional printing may also be facilitated by incorporating large amounts of immiscible fillers (e.g., talc, lactose, microcrystalline cellulose (MCC), starch, magnesium stearate/carbonate, and tricalcium phosphate) in the preparation of filaments. Table 3 also highlights the possibility of adding solubilizers (e.g., Soluplus^®^ or PVP), disintegrants, colorants, and modifiers of the drug release profile (e.g., by combination of polymers).

Melochi et al. [25] have evaluated the suitability of many common polymers (used in pharmaceutical formulations as raw materials for HME filament production) for FDM as well. This work proved that filaments based on insoluble (EC and Eudragit^®^ RL), promptly soluble (PEO and Kollicoat^®^ IR), enteric soluble (Eudragit^®^ L and HPMCAS), and swellable/erodible (HPC, HPMC, PVA, and Soluplus^®^) polymers were successfully produced and may be employed for the 3D printing of disks. In this work, a trial-and-error approach was used until appropriate polymeric formulations for both hot-melt extrusion and printing were obtained. First, the researchers swapped the standard spring with one of lower stiffness in order to decrease the strength applied and minimize its impact on more sensitive formulations. Furthermore, the addition of specific amounts of plasticizer was also tried as an option whenever rupturing or wrapping issues of filaments had arisen. The amount of plasticizer ranged from 5% to 20% for all formulations, except for those composed of PEO and HPC, which did not contain TEC; Eudragit^®^ formulations had the largest amount of plasticizer [25].

Formulations of cellulosic derivatives (HPMC, HPC, HPMCAS, and EC) were prepared at extrusion temperatures ranges from 160–180 °C and printing temperatures between 180–200 °C; PVA filaments were prepared at a higher extrusion (190 °C) and printing temperature (225 °C); on the other hand, formulations with PEO, Soluplus^®^ [polyvinyl caprolactam-polyvinyl acetate-polyethylene glycol graft copolymer (PCL-PVAc-PEG)] and Eudragit^®^ RL exhibited the lower extrusion temperature (65–120 °C) and the printing temperature was below 200 °C for all formulations. When used as drug permeability barriers, such disks were promptly soluble (e.g., Kollicoat^®^ IR and PEO), swellable/erodible (HPC, HPMC, PVA and Soluplus^®^), slowly-permeable insoluble (EC and Eudragit^®^ RL) and gastro-resistant layers (Eudragit^®^ L and HPMCAS), according to the polymeric matrix composition [25] and amenable to address main drug release profiles required in pharmaceutics. This example illustrates the need for both adjusting formulation and processing parameters for better extrudability and printability. Table 4 gives an overview of the works done integrating HME and FDM, the type of polymers and equipment used in the processes, and the dosage forms produced. Special emphasis is given to the HME and FDM critical parameters’ set-up, which are fundamental for success and must be carefully controlled.

### 4.1. Polyvinyl Alcohol

**Figure 4:**
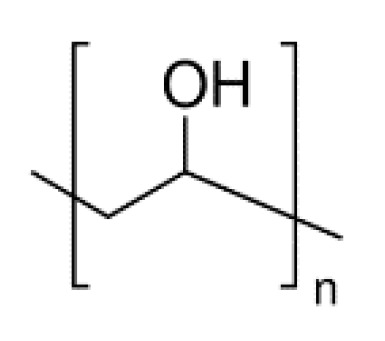
Chemical structure of polyvinyl alcohol (PVA).

Polyvinyl alcohol (PVA) is a water-soluble crystalline support material (helping the printing of specimens with complex geometries) with a good adhesion to several polymeric materials and thermal stability. PVA (Figure 4), which is obtained from polyvinyl acetate (PVAc) through alkaline hydrolysis, is easily degradable by biological organisms and is atoxic. PVA is considered a safe pharmaceutical excipient (already used in many drug delivery systems and listed both in the United States [95] and European Pharmacopoeias [96]), which, together with the thermoplastic properties, makes this polymer an appropriate matrix for fabricating good drug-loaded filaments that, in the end, will provide FDM 3D-printed tablets with the required quality attributes [27,42,43,59]. In fact, PVA has already been used for the production of drug-containing filaments, both by the soaking method (resulting in very low amount of drug loaded (1750 ± 230 µg/g [97] and 0.06–0.25% *w/w* [45]) or HME [17,55], when drug loading can reach up to 40% [43,51,62,93]. In many cases, a commercial spool of PVA has been simply cut into small pieces (~2 or 1 mm), milled, sieved (1000 mesh) and hot-melt extruded, though reaching a maximum drug loading of 9.5% *w/w* [50].

Depending on the degree of hydrolysis of the acetate groups, the melting point of PVA may range from 180 °C (partially hydrolyzed) to 228 °C (fully hydrolyzed). There has been indication that the amount of PVA in the matrix affects the release rate of the drug loaded [50], governing drug release behavior.

Saviano et al. [43] have conducted an experiment to systematically evaluate the effect of PVA particle size on mixing-extrusion-printing steps. As drug adhered completely to the surface of smaller polymer particles (250–600 µm), greater homogeneity of both filaments and printlets (3D-printed tablets) was promoted, resulting in better printability and drug content. Moreover, irrespective of drug content in the filament used for printing, printlets showed similar drug release profiles.

Goyanes et al. [50] have also established the possibility of combining HME, FDM 3D printing, and film coating for the manufacture of PVA-based modified-release budesonide solid dosage forms. Budesonide was loaded into PVA filaments using HME, capsule-shaped tablets (caplets) were printed by FDM, and these were then over-coated with a layer of enteric polymer. However, budesonide adhered to the walls of the extruder (hopper and barrel) during extrusion and components were irregularly extruded, resulting in low drug loading. The problem was solved by improving mixing and altering shear force and pressure within the barrel. The 3D-printed caplets started to release drug in the mid-small intestine, and release continued in a sustained manner during transit in the distal intestine and colon.

Li et al. [60] also combined HME and FDM to prepare a drug delivery device intended to treat diabetes. Glipizide was loaded onto PVA by HME; filaments were used to feed a dual extruder FDM 3D printer to produce a device (DuoTablet) with two chambers where a tablet was embedded within a larger tablet. The two chambers contained different doses of glipizide, and the outer layer released the drug quickly, while the inner layer presented a slower release. This work has shown the possibility of multi-dose administration of a single drug.

Jamroz et al. [90] 3D-printed orodispersible films of aripiprazole using PVA for the development of customized paediatric dosage forms. With a similar goal, a pediatric formulation of baclofen [55] using PVA and 10% sorbitol as a plasticizer, was printed into minicaplets of different sizes, infill percentage and patterns. The minicaplets were produced with precise doses and the required release profile by resorting to minimal excipients, as required in pediatrics.

Many other authors [51,52,54,62] have used PVA as a matrix polymer to obtain different drug release profiles. For instance, a group of researchers [52] proposed 3D-printed floating devices combined with a commercial amoxicillin capsule (Sia-Mox^®^). These devices were manufactured using FDM followed by a thermal crosslinking of PVA. Results showed that the fluctuation time of the devices incorporated into the Sia-Mox^®^ was significantly longer than that of the commercial Sia-Mox^®^ capsule and that the fluctuation time increased upon crosslinking.

As discussed before, a combination of drugs in the same dosage unit (polypill) can improve medication management and patient compliance. With this mindset, a polypill was 3D printed by FDM [51] for hypertension treatment. PVA filaments contained four model drugs (lisinopril dihydrate, indapamide, rosuvastatin calcium and amlodipine besylate). The impact of the tablet’s architecture was explored using multilayer and unimatrix structures. A new approach to the use of distilled water as a “temporary co-plasticizer” was reported, and water has been found to significantly reduce extrusion (from 170 °C to 90 °C) and 3D-printing (from 210 °C to 150 °C) temperatures, with a consequent reduction in the thermal stress of chemicals. X-ray powder diffraction (XRPD) indicated that lisinopril dihydrate and amlodipine besylate maintained their crystalline form, while indapamide and rosuvastatin calcium were essentially amorphous in the tablets. Release profile from the multilayer polypills, was dependent on the position of the drug in the multilayer. In addition to the multilayer architecture, which offers greater flexibility in dose adjustment and a good approach to meet the expectations of patient-centered therapy, the possibility to modulate the release of drugs with different physical and chemical characteristics was proven.

Another group of researchers [62] used PVA for the production of filaments via HME using haloperidol and carvedilol as models. After screening several plasticizers, sorbitol was selected to increase the extrudability by melting the PVA. To determine whether a solid amorphous dispersion (ASD) was formed, which would facilitate the rapid and pH-independent dissolution, the miscibility of the drugs in PVA was tested, with and without added sorbitol. The drug release from physical mixtures, crushed extrudates, and printed tablets was established. Filaments containing 10% and 20% drugs required an extrusion temperature (HME) of 180–190 °C (reduced to ≈150 °C by adding 10% plasticizer) due to the high melting point and high melting viscosity of PVA. However, a temperature of 210 °C was necessary for 3D printing. Miscibility of drugs in PVA was determined as ≈20% for carvedilol and <10% for haloperidol. The polymer matrix ensured a complete drug release from 3D- printed tablets (10 and 20% carvedilol and 60% infill) in around 45 min, at pH 2 and 6.8. In spite of the fairly rapid dissolution rate, the high handling temperatures required and the low drug miscibility in the polymer constitute major drawbacks of this approach.

Matijasic et al. [54] designed and produced modular capsules. FDM was used to produce with PVA a concentric compartmentalized capsule and a modular capsule with different membrane thicknesses. Printed capsules were filled with a blend of dronedarone hydrochloride and ascorbic acid powders and dissolution rate tested. In vitro studies were performed at different pHs to investigate the performance of the capsules. The time delay of the modular capsules in acid medium (0.1 M HCl) was related to the thickness of the membrane. The inner part of the compartmentalized capsule was gastro-resistant for 2 h; thus, it was considered suitable for drug delivery to the small intestine. Results also show that both capsules can be used as modular devices to deliver drugs, when a delayed release is required, or for the release of two different active ingredients.

The immense possibility of using PVA for the production of drug delivery systems by 3D printing was reviewed in this section. It was demonstrated that the extrusion and printing temperatures can vary according to the use of a plasticizer and that drug release can be tailored depending on the combination PVA/plasticizer/drug, thus allowing innumerable possibilities of use.

### 4.2. Polyvinylpyrrolidone

**Figure 5:**
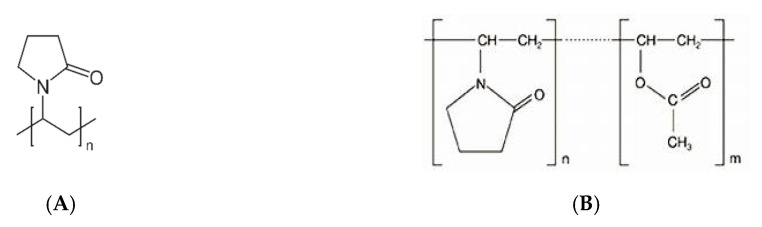
Chemical structure of water-soluble polyvidone (PVP) (povidone; (**A**)) and water-insoluble PVP vinyl acetate copolymer (copovidone; (**B**)).

PVP, commonly called polyvidone or povidone, is a linear non-ionic polymer made of repeating units of the monomer N-vinylpyrrolidone (Figure 5A). PVP is a high polarity/proton acceptor, amphiphilic polymer, which is soluble in water and other polar solvents. The Tg of PVP relates directly to its molecular weight, attaining a plateau at about 177 °C (Table 2), for a molecular weight of 100–150 kDa.

K-values, assigned to different grades of PVP, relate to the degree of polymerization, average molecular weight, and intrinsic viscosity [98]. Varying the degree of polymerization and crosslinking, two different vinyl polymers are obtained: povidone and crospovidone [98]. Povidone (e.g., Kollidon^®^12PF, Kollidon^®^17PF, Kollidon^®^25, Kollidon^®^30, Kollidon^®^90) is water-soluble and presents a molecular weight ranging from 8 kDa to 10 kDa. It is a widely used pharmaceutical excipient employed as a solubilizing agent and crystallization inhibitor [99]. There is also copovidone (e.g., Kollidon^®^ VA64), which is insoluble in water and obtained by physical crosslinking of PVP with a bifunctional monomer (PVP vinyl acetate copolymer). Copovidone presents a molecular weight above 70 kDa [99,100], and it is widely used in pharmaceutical formulations as a direct compression, tablet disintegrant, film-forming and taste-masking excipient, also known for its suitability for HME [100].

PVP is a pharmaceutical grade polymer commonly used in FDM (Table 3 and Table 4). Okwuosa et al. [58] aimed to use this polymer for instant on-demand 3D printing of immediate-release tablets. This is especially relevant since most of the polymers are not entirely appropriate for the formulation of immediate release dosage forms, which are the most prevalent (≈70%) oral formulations. In this study, an optimized ratio of a powder mixtures consisting respectively of PVP, TEC (plasticizer), talc (filler) and API (theophylline or dipyridamole) (10%, 12.5%, 27.5%, and 50%) was used. The physical mixtures of PVP:TEC:Talc: API were gradually homogenized and extruded at 90 °C, at a torque of 0.4 Nm. Filaments were kept in sealed plastic bags at room temperature until printing. Noteworthy is that the presence of a thermostable filler/lubricant (e.g., talc) was very important to enable the fabrication of 3D tablets. In fact, when filaments of PVP alone (no filler) were used to feed the printer, it was impossible to fabricate a structure due to the poor flow from the hot nozzle of the printer; the introduction of talc in the filament allowed formation of a stable structure and rapid solidification of the specimen. The 3D-printed tablets, loaded with dipyridamole or theophylline, demonstrated excellent mechanical properties, acceptable in-batch variability and an immediate release pattern, in vitro. The possibility of lowering the printing temperature using this hydrophilic polymer disclosed the potential of adapting this 3D-printing technology to a wider spectrum of drug molecules and to common use immediate release dosage forms [58].

With a similar purpose, Kollamaram et al. [47] explored the application of the PVP-vinyl acetate copolymer (Kollidon^®^ VA64) and PVP (Kollidon^®^ 12PF) as the main matrix-former polymers to accommodate thermolabile and low-melting temperature substances intended for immediate drug release. Ramipril was used as a model drug since it requires dose flexibility and could benefit from using lower printing temperatures, due to its low melting point (~109 °C). Kollidon^®^ VA64, PEG 1500, mannitol, ramipril and magnesium carbonate (65%, 20%, 10%, 3%, and 2% *w/w*, respectively) were manually homogenized and then extruded. Filaments were also formulated by replacing 50% (1:1) and 40% (3:2) of Kollidon^®^ VA64 from the above formulation with Kollidon^®^ 12PF; mixtures were extruded at 65 °C. Filaments were kept in a vacuum desiccator before printing. Ramipril (8.9 mg) tablets were successfully printed at 90 °C, regardless of the formulation. The use of Kollidon^®^ VA64 and Kollidon^®^ 12PF in low-temperature FDM was further validated using 4-aminosalicylic acid (4-ASA), which remained stable [45]. These results contrast with previous findings obtained in an earlier study, in which 4-ASA underwent degradation during the printing process [45]. Therefore, the above-mentioned studies demonstrated that both selection of excipients and application of new polymers may positively contribute to broaden the scope of FDM printing usage, namely by reducing drug degradation, as a consequence of thermal heating.

Another work [68] also explored the potential of PVP (and other polymers), for FDM printing of the immediate release tablets, at low processing temperatures. In this study, pure polymer filaments were firstly used to infer the printing characteristics of each material. Pantoprazole was then included in the polymeric formulation, when the printing of the pure polymer filaments occurred favorably. In turn, if the printing temperature was far too high (>100 °C) or if solids recurrently clogged the nozzle, these polymeric formulations were disregarded. Based on that, Kollicoat^®^ IR [a graft copolymer comprised of polyethylene glycol and polyvinyl alcohol (PEG:PVA)] and PEO 100 kDa were excluded due to the first and second reasons, respectively. Blockage of the nozzle may be related to a high solid content, irregular distribution of particles or the formation of solid agglomerates which can surpass the diameter of the 3D-printer nozzle. In fact, filaments with Kollidon^®^ CL (as disintegrant) often clogged the nozzle due to the incomplete melting at the extrusion temperature (41–43 °C), with subsequent formation of large particle agglomerates. As a consequence, only poly-vinylpyrrolidone (PVP K12), polyethylene glycol 6000 and 20,000 (PEG 6000; PEG 20,000), Kollidon^®^ VA64, and poloxamer 407 were successfully extruded to produce drug-loaded filaments and printed to tablets containing the thermo-sensitive drug. The extrusion temperature was closely controlled along the heating elements (very slight temperature increase in a defined period of time before starting the extrusion), in order to ensure homogeneous heat distribution. Nevertheless, when compared with PEG-containing polymers printed at temperatures from 54 °C to 60 °C, PVP-based tablets were printed at higher temperatures (between 78 °C and 87 °C for different polymeric formulations). Pantoprazole sodium was incorporated into all polymers at a standard drug load of 10% (*w/w*) and, for PVP K12, at up to 30% drug; filaments were successfully extruded. To find the optimal polymer-plasticizer ratio, several PVP formulations had to be extruded. This work corroborates the crucial role of an appropriate level of plasticizers for a suitable 3D-printing process, as the pure PVP-based formulations could not be printed due to the breaking of filaments inside the printer head as a result of its brittleness. As a consequence, extrusion and printing speed were reduced, and drug content sometimes was not uniform. In turn, over-plasticization of filaments (e.g., formulation with 20% of TEC) may lead to coiling of the material because of its stickiness. Another finding of this study was the filament deliquescence, (particularly for high content of plasticizer in the polymeric formulation) throughout the storage period. For this reason, the authors recommended an immediate printing after extrusion or, alternatively, a storage at lower temperatures to minimize the occurrence of such deformation events [68].

Okwuosa et al. [56] also used PVP for the production of gastric-resistant tablets for delayed release dosage forms. For this purpose, tablets were engineered by employing a range of shell-core designs using PVP in the core and a methacrylic acid co-polymer (Eudragit^®^ L100-55) in the shell. For the preparation of the core structure, PVP (matrix polymer), TEC (plasticizer), talc or tribasic phosphate sodium–TBP–(fillers) were mixed in with drugs (theophylline, budesonide and diclofenac sodium) in different ratios (Table 3) and then extruded under the conditions given in Table 4. The preparation of the shell involved the mixture of Eudragit^®^ L100-55, TEC and talc (50%, 16.67%, and 33.33%) at 135 °C for 5 min and the extrusion at 125 °C by HME. Replacement of talc by TBP, as a filler, allowed a much faster dissolution of the API at the intestinal region. The modification in the drug’s release profile was related to the alkalizing nature of TBP, which led to an increase in the local pH with improvement of water absorption by the core structure, thus accelerating the dissolution of the shell layer and, accordingly, faster release of theophylline. Despite this, talc was deemed as the preferable filler since TBP was associated with API degradation. In fact, a high-pressure liquid chromatography (HPLC) analysis demonstrated a significant decrease in the drug content; thermogravimetric analysis (TGA) also unveiled a decline in weight when TBP was used. Tablets demonstrated gastric-resistant properties and a pH-responsive drug release pattern in both phosphate and bicarbonate buffers.

### 4.3. Cellulose-Derived Polymers

Cellulose is a polysaccharide composed of linear, variable length, chains of 1-4-linked β-d-anhydroglucopyranose units, which are covalently linked via acetal functions between the equatorial –OH group of C4 and the C1 carbon atom. Cellulose is a highly hydrophilic polymer, although it is water insoluble due to the high degree of crystallinity and structure. In fact, it comprises tough intramolecular and intermolecular bonding (e.g., hydrogen bonds) between the polymeric chains, which are responsible for its water insolubility. For these reasons, cellulose is typically modified to form water-soluble derivatives, such as esters or ethers [101].

**Figure 6:**
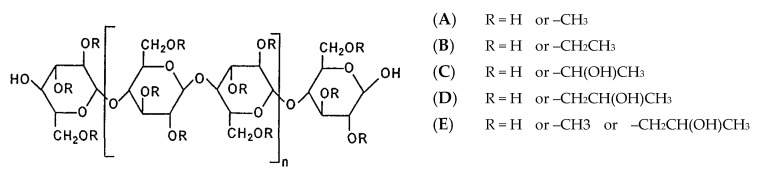
Chemical structure of cellulose ethers derivatives—methylcellulose (**A**), ethylcellulose (**B**), hydroxyethylcellulose (**C**), hydroxypropylcellulose (**D**), and hydroxypropylmethylcellulose (**E**).

Cellulose ethers are a category of polymers formed by the linking of cellulose to alkyl substituents, such as methyl, ethyl, and propyl groups. As such, some of the most commonly used cellulose esters in the pharmaceutical area are methylcellulose (MC), ethylcellulose (EC), hydroxypropylcellulose (HPC), hydroxyethylcellulose (HEC), and hydroxypropylmethylcellulose (HPMC), whose structures are shown in Figure 6.

Among other applications, cellulose-based polymers are currently used because of their capability to stabilize suspensions and emulsions, form films in coating formulations, and act like bonding and thickening agents. Also, these compounds are particularly useful for oral drug delivery systems due to their resistance to hydrolysis throughout the gastrointestinal tract [101,102]. The Tg of these polymers depends strongly on the structure of the cellulose ethers (Table 2). In general, increasing the degree of substitution of cellulosic hydroxyls, the hydrogen bonding network of cellulose decreases (especially when the substituents cannot form hydrogen bonds) and, as a consequence, Tg also decreases [103].

Recently, these cellulose-based polymers were investigated in a work by Zhang et al. [86], which provided a deeper knowledge about their properties and respective influence on release profile of the incorporated drugs. In this study, researchers developed a standard guide that assists the development of suitable 3D-printing filaments, based on their suitability for processing. These data were complemented with the physico-chemical and mechanical properties characterization, using the Repka-Zhang model [86]. This study tested both pure polymeric formulations (HPC EF, HPC HF, HPMC E5, HPMC K100M, HPMCAS LG, HPMCAS HG, and EC N14) and drug-based polymeric preparations (30% *w/w* acetaminophen as a model) were blended. Once extruded, the physicochemical characterization of filaments helped to predict their suitability for 3D printing. The addition of the model drug affected positively the extrudability of physical mixtures by the reduction of torque, die pressure and temperature required for the extrusion process. This effect was potentially associated with the miscibility between acetaminophen and cellulose-based polymers, which also revealed a crucial role for the drug release modulation [86]. Among all polymers tested, no issues during the printing process were reported for the formulations with HPMC, and therefore, these polymeric matrices were considered as the best option for FDM 3D-printing technology. The only exception was the HPMC K100M–based formulation, which required higher extrusion temperature (>200 °C) due to its high molecular weight. In this work, an interdependence between the mechanical characteristics of the filaments and extrudability of the physical mixtures was also demonstrated. In fact, the most mechanically resistant filaments were obtained when larger torque and die pressure were applied during the extrusion process.

The next subsections are referred to the most common cellulose-derived polymers used as raw materials in HME coupled to FDM 3D-printing technology.

#### 4.3.1. Ethylcellulose

EC is a cellulose ether formed from the chemical reaction between ethyl chloride and alkali cellulose compounds. According to the level of replacement of ethoxy substituents, several different groups of EC are defined: G-type (44.5–45.5%), K-type (45.5–46.8%), N-type (47.5–49.0%), and T-type (>49.0%). Water insolubility and solubility in a diversity of solvents has been also reported for this polymer [104]. In the pharmaceutical field, EC is frequently used as polymeric matrix in sustained-release drug formulations owing to its insolubility in water. Despite that, granulates produced with EC, typically result in dosage forms with good disintegration capability. Other applications of EC comprise its role as a binder (EC-N and EC-T groups at a concentration of 2–10%), coating, and taste masking agent [104]. EC possesses an excellent thermal plasticity and softens between 135 °C and 160 °C, making it a versatile and powerful tool in extrusion processes [103]. EC-based formulations for use in FDM 3D printing have been explored and are described below.

In a study performed by Zhang et al. [4], controlled-release tablets were prepared by FDM 3D-printing coupled with HME. This work screened the suitability for 3D printing of several pharmaceutical grade polymers (including EC), based on the physicochemical and thermal properties of various HME filaments of those polymers, used in different ratios and combinations. The strategy of this work comprises three sequential steps. First, pure polymeric formulations containing 30% *w/w* of the drug (acetaminophen) were prepared. Based on the outcome of the printing process and three-point bend test, the researchers fabricated binary polymer blends, in order to optimize the mechanical characteristics of the filaments. Furthermore, dissolution properties of the 3D-printed tablets were also enhanced by the inclusion of a super disintegrant (Kollidon^®^ CL-F), which occurred in the last stage of work. Binary [(EC: polymer) at a ratio of (35:35)] and tertiary ((HPMC: polymer: disintegrant) at a ratio of (45.5:19.5:5) or (50:15:5)) blends were prepared with polymers HPMC E5, HPC LF, Soluplus^®^, and Eudragit^®^ L100. All EC-based formulations were well extruded and printed.

An EC-based formulation was also used by Yang et al. [63] for the preparation of 3D tablets with predesigned internal support structure to attain sustained drug release. Ibuprofen (as model drug) and EC polymer together with release modifiers (Table 3) were, at first, mixed and extruded into filaments by HME, at 100–120 °C. Filaments were then printed into tablets by FDM with different printer settings (Table 4). Drug content, release modifiers, printing parameters and modelling influenced the tablets’ printability and drug release behavior. An optimized and complete drug release within 24 h was achieved by adjusting the amount of release modifiers, infill pattern and density, and shell thickness of models.

#### 4.3.2. Hydroxypropylcellulose

HPC is a cellulose derivative resulting from the hydroxypropylation of the hydroxyl substituents through the reaction between alkali cellulose and propylene oxide under specific conditions. Among the HPC properties, its relative plasticity and hydrophobicity, its complete solubility in water and organic solvents (e.g., alcohols and acetone), and its low Tg (111 °C, Table 2; depending of moisture content), which is expected to decrease with increasing moisture due to the plasticizer effect of water, are all noteworthy. At equivalent moisture contents, HPC EF and HF (low and high molecular weight) exhibit similar Tg [105]. The high swellability of HPC, especially for high molecular weight molecules, is another relevant feature that makes it appropriate for controlled drug release kinetics. In fact, HPC can be used to modulate the drug release profile, since it exists in the pharmaceutical market under different viscosity levels and molecular weight grades. The drug release rate depends on the polymer viscosity, which in turn is affected by the temperature. Hence, the rise in temperature causes a reduction of HPC viscosity, and consequently, improves the release of the API. HPC-based formulations for FDM 3D printing have been used in several works as summarized in Table 3.

Goyanes et al. [91] studied the influence of shape, size, and color of different placebo FDM 3D-printed tablets (without drug) on end-user acceptability, regarding preference and ease swallowing. Filaments were prepared from a mixture of HPC (73.75%), mannitol (21.25%, as a plasticizer), and magnesium stearate (5%, as a lubricant) using a single-screw extruder and stored in plastic bags until printing. Three-dimensional-printed tablets were then successfully produced at an extrusion temperature of 140 °C. The researchers evaluated ten selected shapes, four different sizes and nine colors. The printlets were consistent in size, shape and appearance.

HPC has been used as a matrix for the immediate release of drugs. A study performed by Pietzak et al. [26] developed a flexible dose tablet system, for immediate and extended release, using two immediate release polymers (Eudragit^®^ E and HPC SSL) and two extended release polymers (Eudragit^®^ RL, Eudragit^®^ RS) and their 1:1 mixture. Theophylline was used as model drug (melting point of 273 °C). Considering the HPC-based formulation, filaments containing API were prepared by extrusion of a physical mixture composed of HPC SS (polymer), theophylline and triacetin (plasticizer) at a ratio of 46:50:4. Filaments were stored in sealed plastic bags at room temperature before 3D printing digitally designed, capsule-shaped tablets with different dimensions. As expected, thermal analysis indicated that most of the drug was promptly released (<30 min) from caplets. This work highlights that the 3D-printing process delays the release of the selected API because of the drop of surface area of the filament, the modification of the polymeric chain entanglement, and the alteration of drug distribution inside the polymeric material during printing, as well.

More recently, a research group employed HPC in the design of an innovative tablet with unique built-in gaps (gaplets) to optimize the drug release rate [36]. Again, theophylline was used as a model drug; drug, polymer, and plasticizer were intimately mixed at 50:45:5 *w/w* ratios. In addition, selected disintegrants (Ac-Di-Sol^®^, Explotab^®^, Primojel^®^, Polyplasdone™ XL-10) were also included in the drug-loaded polymeric formulations at 5% *w/w* ratio. The uniform distribution of the API within the polymeric chains entanglement was ensured through the gentle drug addiction and also by the consistent material homogenization under specific conditions (minimum 5 min; rotation speed 80 rpm). Before printing, HME filaments were kept in sealed plastic at room temperature, during three weeks after manufacturing. Then, 3D-printed tablets were then prepared by FDM under specific conditions listed in Table 4. Gaplets were constituted by eight gaps due to the presence of 9 blocks linked by 3 bridges. In this work, the experimental design included three groups of tablets containing different block widths (0.5, 1, or 1.5 mm) and, in turn, diverse inter-block spaces within each tablet set.

Researchers used real-time UV-imaging technology to monitor the contact between the cellulose-based 3D-printed tablets and dissolution medium [36]. As expected, HPC exhibited first hydration and swelling effects, with consequent polymer expansion. Afterward, an erosion phenomenon of the polymer surface was observed. Whilst swelling phenomenon leads to the delaying of the drug release owing to the development of viscous diffusion pathways, polymer erosion, in contrast, stimulates the release of the API, consistent with earlier works [70]. Moreover, tablet disintegration and, consequently, API release could be negatively influenced by the swelling phenomenon of the gaplets structure, if the blocks spaces were too thin (0.2–0.4 mm). Since the addition of the disintegrants did not revealed a substantial impact on API release from the gaplets developed, this design approach seems to be more efficient than the conventional formulation alternative of adding disintegrants to accelerate tablet disintegration and drug release. Indeed, the introduction of gaps in the tablet structure seems to have minimized the bulk cellulose-based matrix formation and enhanced drug release by both enabling erosion and maintaining minimal diffusion paths within enlarged repeating units [36].

HPC-based formulations have often been used for pulsatile sustained-release of drugs. At first, the feasibility of using FDM to manufacture oral capsular devices composed of HPC polymer for pulsatile drug release with similar size and shape to Chronocap™ system prepared by injection molding, was evaluated [83]. In this work, HPC filaments were prepared using different conjugation of formulation composition and process parameters (Table 4). Before the extrusion process, HPC polymer was kept at 40 °C for 24 h and then used in a single mixture or combined with PEG as a plasticizer (Table 3). HME was successfully performed at an extrusion temperature ranging from 150 °C (formulation with 10% PEG) to 165 °C (formulation without PEG) and screw speed ranging from 50 rpm (formulation without plasticizer or with 2% PEG) to 60 rpm (formulation with 5–10% PEG). Afterward, 3D printing was carried out from the HPC-based filaments using a printing temperature that was gradually decreased to 180 °C, leading to an adequate equilibrium between melt flowing and thermal stability of the starting material. In summary, a suitable HPC-based capsular device intended for pulsatile drug release was produced. Similar capsule shells with the equivalent formulation were manufactured by injection molding, also having reproducible, reliable results.

Chai et al. [13] applied HPC polymer for the production of printlets for intra-gastric sustained release of domperidone, a dopamine receptor antagonist widely used in the treatment of gastroparesis and other conditions causing chronic nausea and vomiting. It was chosen as a model drug to investigate the potential of floating and sustained-release in increasing its oral bioavailability and reducing the administration frequency. Therefore, hollow-structured tablets (less dense and capable of floating) were developed for intra-gastric drug delivery. Domperidone-loaded HPC filaments constructed the outer shell; to produce low-density tablets infill was kept low. The formulation and HME and FDM parameters of filaments are given in Table 3 and Table 4, respectively. In short, HPC, drug/HPC (10:90), and BaSO_4_/drug/HPC (10:10:80) were continuously extruded at 145–150 °C and a screw speed of 20–25 rpm. The filaments were then printed at 210 °C into hollow structured tablets through changing the shell numbers (1, 2, 3, and 4) and the infill percentage (0%, 10%, 20%, and 30%). Drug-loaded hollow tablets were successfully fabricated and the buoyancy of tablets was closely related to their densities. Tablets (shells ≥3 and infill ≥20%) exhibited a relative higher density (≥0.9 g/cm^3^) and sunk to the bottom of the dissolution vessels, while optimized formulations (two shells and 0% infill) were less dense (~0.77 g/cm^3^) and floated. Due to the rigid shells produced by melting deposition, HPC polymer chains dissociated slowly and thus produced the floating sustained-release effect. Physical characterization revealed that domperidone distributed in the HPC had been converted into a solid dispersion with the application of elevated temperatures [13].

In another study, Kimura et al. [66] also developed FDM 3D-printed tablets, which achieved both floating and zero-order sustained-release. For this purpose, they designed a hollow structure enwrapped by outside shells with a variety of thicknesses. Itraconazole was used as a model drug, and both HPC and PVP polymers were used to prepare 3D-printed tablets. While HPC polymeric matrix was associated with the compact structure of tablets and their extended floating period; the PVP was used in order to enhance itraconazole solubility, which is paramount for bioavailability. The authors proposed that PVP concentration affected the mechanical properties of filaments, since low content of PVP originated malleable filaments, while a high proportion of this component produced brittle filaments. The study was carried out using the formulation containing 20% of API, 65% of HPC and 15% of PVP, because it showed suitable feedability and printability. Thermal analysis revealed that there was no degradation of polymers and drugs, at temperatures below 185 °C (slightly higher when compared with melting point of API in order to allow amorphization). The temperature used was strictly controlled, since very high temperature could make 3D-printing impracticable and cause degradation of formulation components. Furthermore, physicochemical characterization and in vitro drug release profile of floating tablets were also assessed. Results evidenced the amorphous state of the API in the 3D printed tablets, and also, its solubility improvement after the printing process. Also, the drug dissolution and floating tests demonstrated the interrelationship between the outside shell thickness of tablets, the drug dissolution, and the floating time. For instance, there was a retardation of drug release and an extension of floating time whenever the outside shell thickness increased.

Recently, HPC was used by Dumpa et al. [71] for the development of a novel core-shell gastro-retentive tablet intended for floating and pulsatile drug release. HPC and EC-based filaments were used to print the shells. The immediate-release core tablet prepared through direct compression was composed of theophylline, croscarmellose sodium, MCC, and magnesium stearate in a 57:8:34:1 ratio. Prior to FDM 3D printing, the filaments were kept in a desiccator to minimize the influence of moisture content on softening and squeezing of the filaments between the feeding gears of the printer. In fact, the molten material was unable to be pulled through the heater, and an irregular flow of materials along the printer nozzle was observed. These phenomena were directly related with the influence of moisture absorption on the filaments’ flexibility. In this work, the tablets presented the same dimensions but dissimilar shell thicknesses, wall (outer shell) thicknesses, and infill densities. Therefore, pure HPC filaments were first used to manufacture the hollow tablets with four types of shell thicknesses (0.8, 1.2, 1.6, and 2.0 mm). Then, tablets composed of three different wall thickness (outer shell) (0, 0.8, and 1.6 mm) and three individual infill (50%, 75%, and 100%) were printed using the filament containing 0.5% EC to evaluate the impact of wall thickness and infill density of the shell on the drug release profile. Herein, they concluded that the current approach could produce high-quality floating tablets, since there was no breaking or squeezing issues during the process [71].

#### 4.3.3. Hydroxypropylmethylcellulose

Hydroxypropylmethylcellulose (HPMC) belongs to the group of cellulose ethers in which one or more of the three-hydroxyl groups present in the cellulose ring have been substituted (Figure 6). HPMC is a swellable, hydrophilic (water soluble), biodegradable, and biocompatible polymer with a wide range of applications in drug delivery [106]. HPMC is also soluble in polar organic solvents, enabling the use of both aqueous and non-aqueous solvents. It has unique solubility properties; it is soluble both in hot and cold organic solvents [107]. The Tg of HPMC varies between 96–145 °C (Table 2; dependent on molecular weight) and the onset of degradation is above 220 °C [103], which makes this polymer an excellent candidate for extrusion-related processes [108].

HPMC possesses increased solubility in organic solvents and thermo-plasticity, as compared to other methyl cellulose counterparts. It forms gels upon heating with gelation temperature of 75–90 °C, which may influence drug stability concerning 3D printing. One of its most important characteristics is its high swellability, which has a significant effect on the release kinetics of the incorporated drugs. Upon contact with water or biological fluid, water diffuses into the device, resulting in polymer chain relaxation with volume expansion; diffusion of the incorporated drug out of the system follows [106].

Siepman et al. [106] reviewed the mathematical models developed to describe drug release from HPMC-based pharmaceutical devices, clarifying the overall drug release mechanism. At the beginning of the process, an abrupt concentration gradient is established at the media/tablet interface when the tablets come into contact with dissolution media. As previously mentioned, the Tg of the system is reduced by the plasticizer action of water; once Tg is reached, the polymer chains undergo transition from the glassy to the rubbery state, so the matrix is transformed into a hydrogel, changing the drug content at the media/tablet interface. Upon contact with water, the concentration gradient supports the diffusion of the drug from the media/tablet interface to the hydrogel and into the dissolution media. In the case of high initial drug loadings, the inner structure of the matrix changes significantly during drug release, becoming more porous and less restrictive for diffusion as drug depletion occurs. This means that the dissolution and diffusion capability of drug within the HPMC polymeric chains is impaired by the large fill density and improved by high porosity.

These mechanisms were later explored by Zhang et al. [4], as previously mentioned in this review, for EC matrices and, in the present section, for HPMC. Filaments of HPMC alone, or in mixture with other polymers (loaded with 30% acetaminophen, as a model), were evaluated for their suitability for HME-coupled FDM 3D printing. Binary (HPMC:polymer; 35:35 ratio) and ternary (HPMC:polymer:disintegrant; 45.5:19.5:5 or 50:15:5 ratio) blends were prepared with other polymers (EC N14, HPC EF, HPC LF, Soluplus^®^, and Eudragit^®^ L100). All HPMC formulations were extruded at 180 °C, which was a higher temperature when compared with other formulations. Here, 3D-printed tablets were produced at 200 °C (extrusion temperature) for all formulations. When compared to directly compressed counterparts, printlets exhibited better mechanical properties and improved drug release profile and aspect.

Concurrently, the same authors [48] fabricated HPMC-based tablets with different designs and API release profile, which aimed at the controlled release of drugs. The polymeric formulations included Benecel^TM^ HPMC E5, Soluplus^®^, and acetaminophen (as a model drug). First, physical mixtures were extruded at 160 °C, at a screw speed of 50 rpm, and then, filaments were used as starting material for 3D printing at 200 °C. The tablet dimensions did not change through the study; however, individual outside shell thickness and core fill densities were varied. This work evidenced the successful manufacturing of drug solid-dispersions in HPMC-based filaments, through an extrusion process, and the production of zero-order controlled release tablets with diverse 3D structures. The authors have concluded that this design is an efficient approach to optimize controlled drug release rates, constituting a better choice, as compared to modification of formulation, polymer, or processing. Due to the dense outside shell structure, a hydrogel barrier that efficiently controlled the drug release rate from polymeric matrix was formed.

In another study [57], HPMC was used to develop gastro-retentive carvedilol tablets. The drug-loaded polymeric formulation comprised carvedilol (20% *w/w*), HPMC (60% *w/w*), Eudragit RS PO (15% *w/w*), and Kolliphor TPGS (5% *w/w*). As previously reported in some studies, the extruded filaments were stored in sealed plastic bags to attenuate the influence of moisture absorption on their quality. Print design parameters were adjusted (shell thickness = 0.6 mm or 0.9 mm, layer height = 0.1 mm or 0.3 mm) and evaluated on release profile and structural integrity of HPMC-based polymeric chains entanglement. Infill (20%) remained constant, with a large internal free volume, nonetheless still giving a matrix intended to assure structural and functional integrity. Physicochemical characterization and thermal and mechanical tests demonstrated the suitability of this HPMC-based formulation.

HPMC-based formulations were also explored for the production of multi-compartment capsular devices meant for oral drug delivery, to allow the association of incompatible drugs or different drug formulations. Maroni et al. [34] designed such a device using HPMC, KIR (Kollicoat^®^ IR), hydroxypropyl methyl cellulose acetate succinate (HPMCAS), and PEG 400 and 8000. The compartments were generated by the association of two hollow structures by means of a joint, which also functioned as a barrier. These hollow structures were prepared under the same extrusion and printing conditions, as previously detailed [25].

Through combination of compartments having wall thickness of 600 μm or 1200 μm, composed of promptly soluble, swellable/erodible or enteric soluble polymers, devices showing two-pulse release patterns consistent with the nature of the starting materials were obtained. Particularly, promptly soluble (e.g., Kollicoat^®^ IR), gastro-resistant (e.g., HPMCAS), and swellable/erodible (e.g., HPMC) compartments were developed, which allowed immediate, enteric, and pulsatile release to be achieved, respectively. By assembling these compartments differently, two pulse release patterns, characterized by one or more lag phases, were obtained [34].

#### 4.3.4. Hydroxypropylmethylcellulose Acetate Succinate

**Figure 7:**
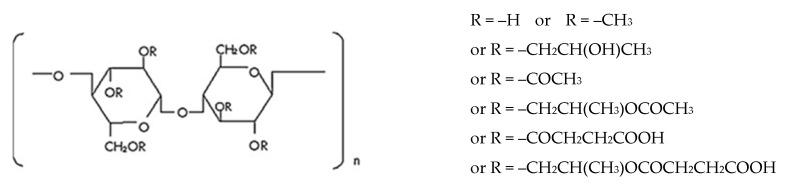
Chemical structure of hydroxypropylmethylcellulose acetate succinate [34,35].

HPMCAS is an enteric coating material developed for both regular enteric coating [109] and sustained release formulations [110]. It was also used in technologies, such as solid dispersions [111,112]. HPMCAS is a mixture of acetic acid and monosuccinic acid esters of hydroxypropylmethyl cellulose (Figure 7); therefore, it is marketed in three different grades depending on the ratio between acetyl and succinoyl groups—L (low ratio), M, and H—with pH thresholds of 5.5, 6.0, and 6.5, respectively [34,65].

HPMCAS filaments have been increasingly used for HME-coupled FDM 3D-printing technology, as indicated by recent studies [34,35], underlining its application on enteric and sustained release of drugs. For instance, Goyanes et al. [49] manufactured 3D-printed tablets (printlets) from three subtypes of HPMCAS (HMPCAS LG, MG, and HG) by both HME and FDM. The composition of the formulations included HPMCAS:drug:methylparaben (plasticizer):magnesium stearate (lubricant) at a ratio of 75:5:15:5 or 40:50:5:5. Drug and excipients were mixed and then extruded using a single-screw filament extruder. Filaments were protected from light and kept in a vacuum desiccator until printing. Two infill percentages were selected (20% and 100%) in order to produce printlets of low and high density. This work corroborates that FDM 3D printing allows the manufacture of delayed-release printlets, without the necessity for an outer enteric coating, along with the customization of the release kinetics as a function of individual characteristics of each patient.

Finally, Scoutaris et al. [65] also used HPMCAS to fabricate chewable tablets with selected shapes articulating HME and FDM 3D-printing technologies in a single process. This approach proved to be particularly interesting for the pediatric population, since HME was effective in masking the taste of bitter APIs (e.g., indomethacin). Furthermore, FDM 3D printing provides the capability to fabricate tablets with a variety of complex shapes, with clear advantages regarding adherence to the medication regimen by children. During this study, HME efficiently dispersed indomethacin within the polymeric chains and provided very good taste masking. Moreover, FDM 3D printing was demonstrated to provide high reproducibility, accuracy and content uniformity of the animal candy shapes produced. This study paved the way for alternative methods to produce palatable pediatric dosage forms.

### 4.4. Acrylates

In this section, the methacrylic acid derivatives present in the formulations as the main matrix-former polymers are discussed.

#### 4.4.1. Eudragit^®^ E PO

**Figure 8:**
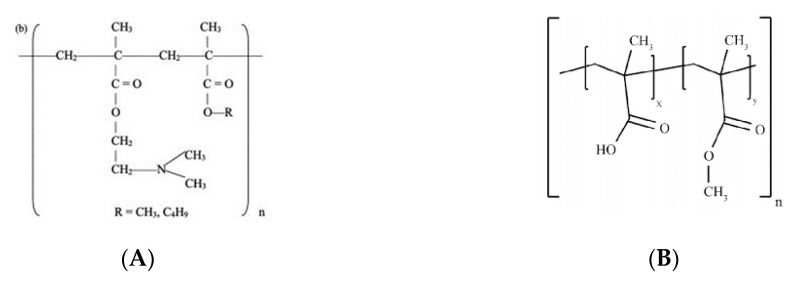
Chemical structure of Eudragit^®^ E (**A**) for immediate release and Eudragit L (**B**) for delayed release [113,114].

Eudragit^®^ E PO (MW ≈ 47,000 g/mol) is an amorphous, cationic polymer carrier. It is a random copolymer consisting of dimethylaminoethyl methacrylate, butyl methacrylate, and methyl methacrylate in a ratio of 2:1:1 (Figure 8A).

Solid dispersions of Eudragit^®^ E PO have been used for various drugs to improve their solubility and bioavailability [115]. Eudragit^®^ E PO is also a suitable carrier for specific delivery to the stomach due to its fast rate of dissolution in the gastric fluid pH, up to a pH of 5.0. Moreover, the insolubility of Eudragit^®^ E PO in neutral and basic solutions may enhance the stability of the drug in a liquid raft forming formulation. The ability of cationic E PO to form interpolyelectrolyte complexes with various anionic polymers was also previously exploited in the design of solid dosage forms for gastrointestinal delivery [29,42,44].

In the context of the present review, Eudragit^®^ E PO was used to print immediate release tablets [29,42]. Sadia et al. [29] used several model drugs in this matrix and investigated the addition of non-meltable filler material to the methacrylic matrix as a means to enhance 3D FDM printing. The impact of (i) the nature of the filler, (ii) compatibility with the 3D printer’s gears, and (iii) polymer:filler ratio in the 3D-printing process, were evaluated. They prepared various filaments containing Eudragit^®^ E PO, TEC (plasticizer; at 5%, 6%, 6.5%, and 10% *w/w*) and a non-melting filler (tri-calcium phosphate–TCP). Based on the on the filler’s stability, compatibility with the gears of the printer head, and rheology of the filament at the printing temperature, the optimal formulation was selected (Eudragit EPO:TEC:TCP (46.75:3.25:50 *w/w*)). This formulation was employed to produce drug-loaded filaments of four model drugs (5-aminosalicylic acid–5-ASA, captopril, prednisolone or theophylline) at 12.5% ratio (drug replaced a portion of the filler). After the two thermal processes (HME and FDM), the drug content slightly decreased—5-ASA (94.22%), captopril (88.53%), theophylline (96.51%), and prednisolone (93.04%). While a fraction of 5-ASA, theophylline, and prednisolone continued crystalline, captopril was in the amorphous state, as indicated by XRPD. Combination of the advantages of thermally stable pharmaceutical polymers and fillers, provides an inexpensive approach to the production of individualized dosage forms on-demand.

Another study conducted by Alhijjad et al. [42] attempted to devise formulation strategies to circumvent these processing problems and provide adaptable release rates for the printed dispersions, through the use of matrix polymer mixtures. The model drug chosen was felodipine, used at a concentration of 10%, and tablets were successfully printed from drug solid dispersions. Tablets contained a mixture of PEG (15%), PEO (15%), and Tween 80 (10%) polymers with Eudragit^®^ E PO or Soluplus^®^. As a reference, to compare the processing of the mixtures developed, PVA was chosen since this polymer has been extensively used in 3D printing. When compared to polymer blends, PVA presents the advantage of having a printing temperature of 150 °C and, at the same time, printing is of excellent quality, using a commercially available 3D FDM printer. Characterization data, using standard techniques, described in Section 5 (e.g., thermogravimetric analysis, differential scanning calorimetry), indicated that the model drug was molecularly dispersed in the polymer matrices. Disintegration of the formulations directly influenced drug release rate, as demonstrated by in vitro dissolution tests. Mixtures containing Eudragit^®^ E PO evidenced bulk disintegration, whereas mixtures mainly constituted by Soluplus^®^ disintegrated strip-by-strip. The authors concluded that it is possible to manipulate the drug release rate in solid dispersions, through the polymer/excipient miscibility, the solubility of the materials in the dissolution medium and the degree of fusion between the printed layers during FDM. These results unveiled possible design approaches to tailor controlled release of formulations.

Researchers also evaluated the in vivo performance of an ovoid oral printlet, containing warfarin (200 or 400 µg); the main matrix polymer was Eudragit E PO [74]. In vivo analysis (rats) using a new approach to UV imaging, indicated that the erosion of the methacrylate matrix was 16.4 µm/min and 15.2 µm/min for the horizontal and vertical planes, respectively. Here, 3D-printed ovoids showed a lower Cmax and a longer Tmax for warfarin, as compared to the liquid formulation.

#### 4.4.2. Eudragit^®^ RL

**Figure 9:**
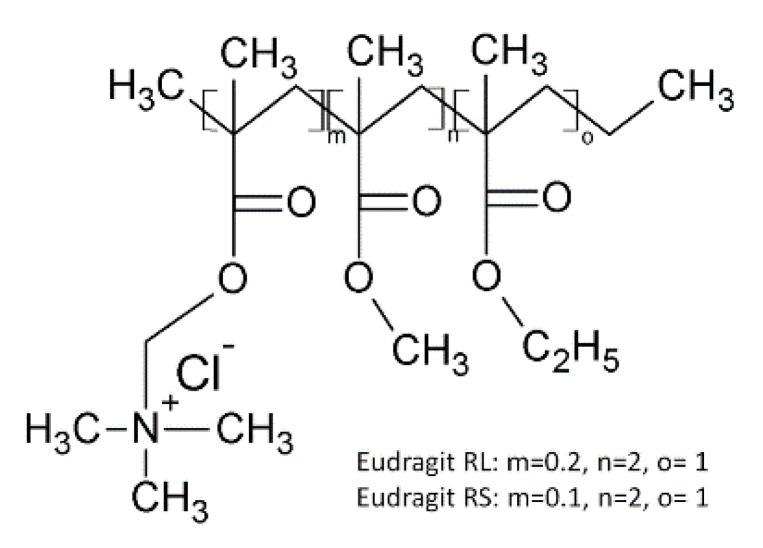
Chemical structure of Eudragit^®^ RL and RS [114].

Eudragit RL, also known as Eudragit Retard L [114] polymer, is a copolymer of poly(ethylacrylate, methyl-methacrylate, and chloro trimethyl-ammonioethyl methacrylate) containing an amount of quaternary ammonium groups between 8.8% and 12% (Figure 9). It is insoluble at physiologic pH values and capable of limited swelling, thus representing a good material for the dispersion of drugs.

Korte and Quodbach [72] proved the possibility of continuously producing large-scale printable drug-loaded filaments by HME. Extruded filaments were strained and cooled down on a conveyor belt, and a combination of mechanical resilience tests were used to predict the filaments’ printability. Eudragit RL was chosen as a sustained release polymer and theophylline (30%) as a thermally stable model drug. Stearic acid (7%) and polyethylene glycol 4000 (10%) were evaluated as suitable plasticizers for the production of 3D-printable filaments. The two formulations were printed in solid ovoid tablets and tested for dissolution profiles. Stearic acid maintained the sustained release properties of the matrix, while PEG 4000 did not. Regarding continuous extrusion, powder feed speed and speed of the filament draw after extrusion predominantly determined the diameter of the filament and, therefore, its mechanical resilience.

The same authors developed a system for sustained drug release, which allows the dose to be adjusted and the drug release to be estimated at the same time [18]. The filaments consisted of Eudragit^®^ RL, theophylline (30%), and stearic acid as a solid plasticizer. Mixtures (>300 g) were extruded using a co-rotating twin-screw extruder, and the powder blend was gravimetrically fed and then extruded with the screw configuration and parameters given in Table 4. In short, depending on the type of polymer and the ratio of the plasticizer, the processing temperature varied between 140–180 °C; during formulation development, a powder feed rate of 5 g/min and a screw speed of 20 rpm were applied. Network structures of diverse densities were printed as a new solid dosage form, considering that the surface/mass ratio was constant. Tests performed using X-ray microcomputer tomography determined the weight, dose, and surface area; these have also demonstrated a linear correlation with the filling density. The filling density did not alter the specific surface area of the network. The tablets printed with denser meshes presented a slower release of the drug, and the Higuchi model was used to predict drug release. However, this model showed limited applicability, as the tablets presented different release kinetics according to the infill. These results are promising, mainly for the customized production of 3D printing sustained release solid forms in the hospital and community pharmacy settings.

To obviate to the shortage of adequate filaments to feed the FDM printers, Melocchi et al. [25] tested various polymers in common use in pharmaceutical formulations, which provided different release behaviors related to the functional application of the polymers used, as already discussed in Section 4. For example, Eudragit^®^ RL resulted in slow permeability to water, and Eudragit^®^ L provided gastro-resistance.

Beck et al. [5] produced a solid dosage form loaded with nano-sized carriers, considered a multi-functional drug delivery system. Polycaprolactone (PCLa) and Eudragit^®^ RL100 were used as the main polymeric compound (64%), with or without mannitol, as a channeling agent. For tablets prepared without the channeling agent, MCC was used instead, due to its low water solubility. TEC was used as a plasticizer and PEG 6000 as a hydrophilic lubricant to facilitate the extrusion process. The filaments were produced using HME. In order to evaluate the influence of the infill percentage, devices from the filament ERL-M were also prepared with 50% of infill. Afterward, the tablets were incorporated with polymeric nanocapsules, as a new platform for the development of oral dosage forms and biodegradable implants. Due to the possibility of tailoring the dose and drug release profile, these dosage forms showed promise in the production of personalized medicines. This approach combined HME coupled FDM 3D printing with nanotechnology to produce novel dosage forms.

The ability of FDM 3D printing to fabricate dosage form containing two APIs was reported by Gioumouxouzis et al. [59]. The double layer dosage form contained two antidiabetic drugs (metformin and glimepiride). Two different polymeric carriers were used to incorporate the drugs to achieve distinct release characteristics. One formulation contained a combination of metformin, Eudragit^®^ RL, and plasticizers (PEG 400, TEC, and citric acid monohydrate) to achieve a sustained-release effect. The filament, which presented the optimum mechanical properties (50% metformin, 35% Eudragit^®^ RL PO, 10% powdered PLA filament, and 5% PEG 400) was used for the preparation of the 3D-printed dosage forms. The second formulation contained glimepiride (2% *w/w*), PVA (80% *w/w*), and mannitol (15% *w/w*) as a plasticizer, and calcium stearate (3% *w/w*) for an immediate release layer. The 3D-printed bilayer dosage form allowed the concurrent intake of two drugs, typically administered at different times of the day, resulting in an easier therapeutic schedule and patient acceptability. This study showed that FDM 3D printing is a promising technique for the fabrication of complex personalized medicines consisting of various APIs, which require different release patterns [59].

Mixtures of other different types of metacrylates (Eudragit RS, RL, E) and HPC were used for the first time by Pietrzak et al. [26]. They prepared five different combinations of polymer, model drug (theophylline 50%) and a plasticizer (TEC). Three-dimensional printing based on FDM proved to be compatible with the drug-loaded filament produced via HME. The linking of 3D printing to the HME process required an increase of 40 °C in the printing temperature, as compared to the extrusion temperature of the corresponding filaments. The majority of theophylline was in the crystalline form in the 3D-printed tablets, as indicated by thermal analysis. A linear relationship between the mass and the printed volume was obtained (R^2^ = 0.9999) and used to digitally control the dose.

#### 4.4.3. Eudragit^®^ RS

Eudragit RS, a polymer of methyl polyacrylate and methyl methacrylate and a low methacrylic acid ester content with quaternary ammonium groups (Figure 9), has been widely used for colon- specific drug delivery systems [25,69]. Researchers [26], have compared Eudragit^®^ RS with other polymers of the Eudragit^®^ range and HPC to tailor the release of a model drug (theophylline), from different matrices, to develop a flexible-dose-dispenser tablet.

Kempin et al. [69] have used Eudragit RS to compare drug release from different matrices, as described below in Section 4.5.

#### 4.4.4. Eudragit^®^ L

Eudragit^®^ L, an anionic copolymer, is based on methacrylic acid and ethyl acrylate in a 1:1 ratio (Figure 8B). This polymer was used by Melochi et al. [25], together with many other polymers, to study the possibility of extruding (HME) and 3D-printing (FDM) several matrices for drug release; the study and results have already been presented in the beginning of Section 4.

### 4.5. Other Polymers

This literature review has also highlighted the applicability of other polymers for HME-coupled FDM technology, such as Soluplus^®^, Kollicoat^®^ IR, PCL, PEO, and ethylene vinyl acetate (EVA), described below.

**Figure 10:**
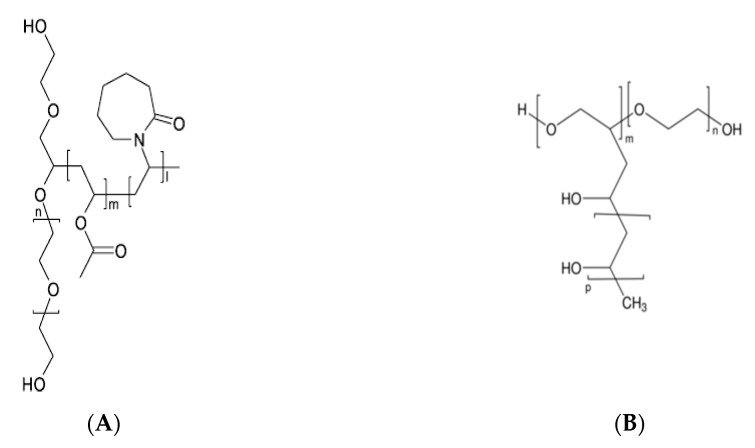
Chemical structure of (**A**) Soluplus^®^ [79] and (**B**) Kollicoat^®^ IR [79].

Soluplus^®^ is a graft copolymer comprised of PCL, PVA and PEG (Figure 10A), which exhibits amphiphilic properties. It is widely used as a matrix polymer for solid solutions and an active solubilizer through micelle formation in water [79]. Due to the low Tg (Table 2) and hygroscopicity, this polymer is also suitable for extrusion processes [116] and it has been investigated for these purpose by a number of researchers [4,43,44,108]. Zhang et al. [4] reported that extrusion of single polymer formulations (Soluplus^®^: acetaminophen; 70:30) was not possible at high temperatures (140 °C) since it melted completely. Conversely, filaments based on Soluplus^®^ were successfully produced [25] and used for printing 600-µm-thick disks.

Kollicoat^®^ IR is a robust yet flexible PVA/polyethylene glycol graft copolymer (Figure 10B). This water-soluble film-forming agent is ideal for manufacturing instant-release coatings for solid dosage forms and for applications such as binding, pore forming and drug layering [79]. An increasing number of papers regarding Kollicoat^®^ IR suitability for FDM 3D-printing, have been published in recent years [34,35,44].

**Figure 11:**
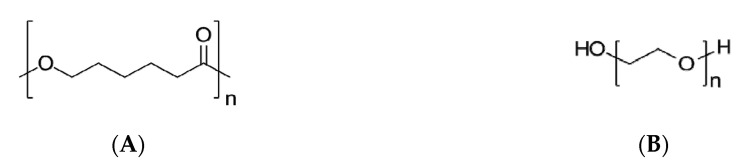
Chemical structure of (**A**) PCLa [117] and (**B**) PEO [118].

A study performed by Kempin et al. [69] considered HME to produce quinine loaded filaments used to manufacture quinine implants. The drug release rates from implants made of different polymers (Eudragit^®^ RS, polycaprolactone–PCLa, PLA, and EC) were compared. Quinine-loaded filaments were produced by solvent casting and subsequent HME; model implants (hollow cylinders, outer diameter 4–5 mm, and height 3 mm) were printed using a standard FDM printer. Production parameters were adjusted for each of the polymers (Table 4). PCLa, a low meting point (60 °C; Table 2) biodegradable thermoplastic polyester (Figure 11A), exhibited the fastest drug release, whereas Eudragit^®^ RS and EC exhibited the slowest. For PCLa, further filaments were prepared with different quinine loads (2.5–25% *w/w*), and thermal analysis proved the presence of a solid dispersion of drug in the polymer at all concentrations tested. Increasing the drug load also increased the overall percentage of drug released to the medium, since nearly the same absolute amount of quinine remained trapped in PCLa at the end of the drug release studies.

Holländer et al. [92] developed T-shaped prototypes of intrauterine devices, containing indomethacin embedded in PCLa, by FDM 3D-printing filaments. The study has demonstrated the possibility of printing medical devices, loaded with different fractions of indomethacin, which can be used in personalized medicine [92].

PEO is a thermoplastic homopolymer of the ethylene oxide monomer (Figure 10B) extensively used in the pharmaceutical industry. However, there is a small number of studies considering PEO in FDM, mostly related to the manufacture of thin oral delivery films combined with additives [93] or added to methacrylates for the manufacture of tablets [42]. In the first work [93], filaments were produced with PEO and ibuprofen or paracetamol as model drugs at 60 °C. Filaments loaded with PVA and paracetamol were also produced, at 130 °C. Furthermore, a filament containing PEO and strawberry powder was used to produce a taste-masking layer. Fast-dissolving oral films were printed at temperatures of 165 °C (PEO) or 190 °C (PVA) with plain or mesh designs. Preliminary studies showed that 30 kDa PEO produced brittle filaments; only PEO 100 and 200 kDa produced suitable filaments. Based on these observations, PVA with high molecular weight was considered. In addition, sodium lauryl sulfate improved drug-release rate from films containing PEO. Starch and super disintegrating agents (i.e., sodium starch glycolate and croscarmellose) were added to the formulations to aid disintegration of films. High-performance liquid chromatography and mass spectroscopy analysis indicated stability of the drug during the film preparation process.

More recently, Isreb et al. [73] developed solid oral forms (tablets) with theophylline considering PEO (molecular weight 100–900 K) as the main polymer in combination with PEG (molecular weight 6 K) as plasticizer. PEO-based filaments yielded easily breakable filaments, thus failing to show good rheological and mechanical properties required for printing. This difficulty was overcome when PEG was included into the formulation, since it functioned as a plasticizer, promoting flow and formation of pores, which increased the release rate of theophylline. Filaments were considered to print tablets with a radiator-like geometry, with interconnected paralleled plates and inter-plate spacing of 0.5, 1, 1.5, or 2 mm. X-ray diffractograms of the filaments revealed the presence of two distinctive peaks at 2θ = 7° and 12°, which can be correlated to theophylline crystals. Filaments made of mixtures of PEO 200–600 K and PEG proved to be mechanically resistant. The addition of PEO of higher molecular weights resulted in filaments with higher shear viscosities (>104 Pas) at the printing temperature and hindered material flow during the FDM 3D-printing process. The geometry of the printed dosage accelerated the release of the model drug through PEO swelling and erosion. This has confirmed the impact of the molecular weight of PEO on printability, showing that the middle range molecular weight (300–400 K) delivered the best dosage forms, whereas lower weight (100–200 K) filaments presented poor mechanical properties, and higher molecular weights (900 k) prevented flow due to high viscosity.

**Figure 12:**
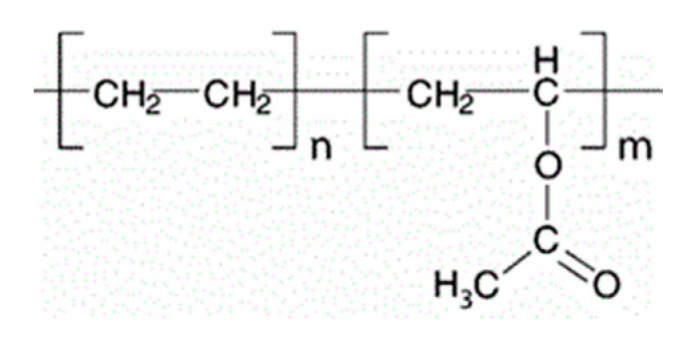
Chemical structure of EVA [119].

Another polymer, ethylene vinyl acetate (EVA, Figure 12), enabled the manufacture of filaments containing indomethacin [64] as intrauterine devices and subcutaneous rods. The work investigated the effect of different EVA grades on the printability of the respective filaments into medical devices. The devices were successfully printed at 165 °C, therefore slightly above indomethacin’s melting point, confirming the ability of EVA to allow the manufacture of both filaments and printed dosage forms.

### 4.6. Commercial Polymer Blends

Polymers blends can be an alternative to obtain optimal extrudability, printability, and drug release, which could be difficult to obtain with single polymers, thus enabling the use of pharmaceutically approved polymers in printing oral solid dosage forms.

Solanki et al. [120] evaluated mixtures of polymers to identify the pharmaceutically acceptable amorphous polymers for FDM of oral dosage forms, for immediate release of haloperidol. Selection criteria were drug release profile and extrusion/printing ability. The water-soluble polyvinylpyrrolidone-vinyl acetate copolymer (Kollidon^®^ VA64) promoted a fast release of the drug in gastric and intestinal pH conditions, although filaments presented poor mechanical properties (high brittleness), preventing printing. On the other hand, filaments obtained with HPMC (Affinisol™ 15cP) and the polyvinyl alcohol–polyethylene glycol graft copolymer (Kollicoat^®^ IR) showed good mechanical characteristics for FDM, but haloperidol was not released in an immediate and regular fashion from crushed extrudates. Consequently, Kollidon VA64, Affinisol™ 15 cP and HPMCAS were mixed to improve the mechanical properties of the filament, making it suitable for 3D-printing. Although HPMCAS is an enteric polymer (dissolves around pH > 5.4) providing delayed release, it was used in a 1:1 ratio with the Kollidon VA64 (highly soluble in water) to improve the mechanical properties and the printed extrudate with haloperidol presented immediate drug release. Both single polymer (Kollidon^®^ VA64, Kollicoat^®^ IR, Affinisol™ 15 cP and HPMCAS) and binary mixtures (Kollidon^®^ VA64 + Affinisol™ 15 cP (1:1); Kollidon^®^ VA64 + HPMCAS (1:1), with or without haloperidol (10% and 20% of polymer blends), were hot-melt extruded (150 °C). Tablets were evaluated for dissolution of haloperidol at pH 2 and pH 6.8 after printing with 10% and 60% infill (210 °C, Makerbot printer). Filaments made of Affinisol™ 15 cP, HPMCAS MG and Kollicoat^®^ IR, alone or in combination with Kollidon^®^ VA64 (1:1 *w/w*) exhibited good mechanical properties for printing. It has been shown that mixtures of polymers with 10% haloperidol were printable due to their appropriate mechanical properties, suggesting that the presence of the drug did not affect printability. The binary mixture HPMCAS MG with Kollidon VA64 has allowed the release of haloperidol at both pHs, but the release rate was slow (120 min were required to release the drug). By opposition, the release of haloperidol from tablets made of Kollidon^®^ VA64 and Affinisol™ 15 cP was immediate and complete, regardless of the pH; this was considered the best system both for printing and fast drug release.

Another example of the advantages of the polymer blends was provided by Kollamaram et al. [47]. As previously reported in the present review (Section 4.2), they used Kollidon^®^ VA64 combined with Kollidon^®^ 12PF to produce immediate-release tablets loaded with thermolabile and low-melting temperature drugs. Other extrudates were manufactured by replacing Kollidon^®^ VA64 by Kollidon^®^ 12PF, keeping the other components of the formulation. The new formulation delivered brittle filaments, which, although obtained at a lower temperature (65 °C), were impossible to print. This was attributed to the lower molecular weight polymer (Kollidon^®^ 12PF) preventing the long range establishment of bonds between the polymer chains, critical for the tensile strength of the filaments. Consequently, authors had to combine Kollidon^®^ VA64 with Kollidon^®^ 12PF resulting on improved filament flexibility and ease of extrusion.

The use of polymer blends to achieve the required release rate of a drug from a filament and printed dosage form with good processability, has attracted the attention of other researchers [4,67]. For instance, PLA and HMPC-based formulations were used to modify the release of nitrofurantoin from a 3D-printed tablets with different geometries for flexible dosing and precision medication [67]. Different mixtures of nitrofurantoin (5% *w/w)*, HPMC (Metolose^®^) at 20% or 40% *w/w,* and PLA were produced. The PLA–HPMC–API combination has been successfully extruded, but HPMC degradation temperature (which degrades at a much lower temperature than PLA) was an issue when setting the processing temperature of the blend. Extrusion temperature was therefore adjusted for each formulation to guarantee HPMC integrity and allow the flow of the blend. Overall, a temperature between 190–200 °C was used in all formulations. Nitrofurantoin was shown to be stable throughout the processes by thermal analysis. Rheological analysis, on the other hand, has shown that the flow of the mixtures was related to the fraction of undissolved particles in the formulation. Dissolution studies have shown that the release of nitrofurantoin was dependent on the fraction of Metolose^®^ present, with higher drug amounts released with higher HPMC fractions in the formulation [67].

Felodipine in solid dispersions of blends of PEG/PEO and Tween 80 with either Eudragit E PO or Soluplus could be printed [42]. As previously discussed (see Section 4.4.1), PVA was used as the reference polymer because of its excellent thermoplasticity and FDM printability. On the one hand, although Eudragit^®^ E PO and Soluplus^®^ both have been widely used in pharmaceutical HME, due to their good thermal stability and extrudability, they are not FDM printable on their own. Therefore, due to low melt viscosity of PEG, this additive was considered to improve the flowability of the blends. PEG provides mechanical flexibility to the filaments whereas Tween 80 acts as a plasticizer which, by decreasing the Tg of the polymer, promotes a decrease on the processing temperatures and as a solubilizing agent, useful for low water solubility drugs. For Eudragit^®^ with a higher viscosity and lower Tg than Soluplus^®^ (Table 2), PEG had to be used in higher fractions. The addition of felodipine to the Eudragit^®^ based formulations promoted a decrease on the polymer Tg improving the printability (e.g., the torque in the extruder was reduced) enabling printing. On the contrary, for the Soluplus^®^-based blends, the plasticizing effect of the drug was not significant, and it was probably masked by the higher fraction of Tween 80 present in these blends. It follows that the release rate of felodipine in the printed dosage form could be adjusted based on the modification of its apparent solubility and ability of the polymer to melt.

Recently, Ilyés et al. [57,121] demonstrated that inclusion of a polymethacrylate derivative in HPMC formulations can improve its processability and the range of temperatures and torque required for processing. This modification of viscosity is suitable for the production of HME filaments that will be later used in FDM, due to the shear thinning and improved mechanical properties provided to the blend. The benefits of the addition of Eudragit RS PO are not only limited to the printing capacity of the filaments but also to the final characteristics of the tablets, such as hardness, which was positively influenced, and the acidic resistance of the tablets providing an enteric effect and extending gastric residence [57].

The combination of different polymeric matrices and the construction of multi-layer structures also have the potential of employing FDM 3D printing on the modulation of drug release. As previously mentioned, Okwuosa et al. [56] developed gastric-resistant tablets with a shell-core structure composed of different polymers—PVP for the core, and Eudragit L100–55 for shell building. Therefore, authors have shown the potential of employing FDM 3D printing to provide flexibility on designing dosage forms with the required release profile of drugs, in contrast to traditional technologies.

Goyanes et al. [50] have used a dual-extruder FDM 3D-printer to fabricate two different capsule-shaped oral drug delivery devices (caplets). They were printed in two different ways: a multilayer device versus a two compartment caplet. The multilayer device was made of two adjacent layers containing different drugs, whereas the two-compartment caplet was made of a smaller caplet embedded in a larger caplet (Duocaplet), with a different drug in each compartment. In this study, PVA-based filaments were loaded with paracetamol or caffeine. The release of either drug has shown that for the multilayer device, drug release rates were similar for both drugs. However, if one layer has a higher drug loading than the other, the release rate will be faster for the one with the higher drug loading. For the dual caplet, the drug on the external layer was released first. The time lag of the release of drug in the inner caplet depends on the characteristics of the external layer. The study emphasized the effect of the design of the dosage form on the release rate of different drugs.

## 5. Characterization of Filaments and 3D Dosage Forms Produced by HME-Coupled with FDM

As discussed in Section 3, although both HME and FDM employ thermoplastic polymers, finding a raw material that meets the basic requirements of both techniques may not be straightforward [23]. The extrudability of the raw materials to form a filament with a uniform diameter and proper thermal and mechanical properties, which affect its rheological behavior while printing, are prerequisites for successful printing and must be carefully controlled. Printed dosage forms are often left to stabilize for a period of time, prior to analysis.

In fact, to achieve successful printing, first, the filament should be able to feed the printer to enable a continuous operation with high throughput. It is important that the dimensions, the stiffness of the filament and viscosity of the molten polymer are appropriate to the adequate functioning of FDM, avoiding breakage or coiling of filaments during printing [23,122] and printer jamming. The diameter determines the feed rate to the heating end, and variations in diameter can result in inconsistent printing that may lead to failure of the process [41]. The balance between the filament’s stiffness and brittleness is critical for printing, since the filament must bend with a minimal deformation in the feeding system and, at the same time, present a minimal hardness to endure compression and tensional forces applied to filaments by the gears feeding the printing head [15,72]. The viscosity of the solid and softened filament also affects its behavior. High viscosity prevents the softened material to flow through the nozzle, whereas low viscosity increases the printing rate preventing proper deposition of the material. In the deposition zone, the printed specimen’s properties are dependent on the flow behavior of the printing material, the cooling and adherence to the previous layer, or to the printing-bed, maintaining thickness of the layers and avoiding the formation of air bubbles or agglomerates of dispersed substances in the filament [123]. A low viscosity of the softened filament at the printing conditions is important for spreading uniformly on the surface of the previous layer, promoting appropriate adhesion between layers, and enabling bridging without flowing as a liquid [120]. Plasticizers (including the drug and water, which may act as plasticizers) can expand the processability window [123]. Poor adhesion and layer coalescence lead to inconsistencies in the structure of the printed specimens (e.g., points of failure, poor performance, and geometrical discrepancies), whereas proper adhesion promotes a strong layer-to-layer bond, providing high mechanical toughness to the printed tablet [81]. Higher printing and print-bed temperatures (within limits) decrease the melt viscosity promoting coalescence of materials due to the increased polymer chain mobility and intermingling between layers [41]. Afterward, fast cooling is required to increase the viscosity of the printed layer for shape retention of the specimen, upon increased weight of printed layers [124]. The latter can be affected by the direction in which the layers are deposited, impacting on the strength of the bonds between them [125]. Overall, materials must have specific mechanical, rheological and thermal characteristics [15] to enable printing. These, together with the main characterization techniques available, are briefly reviewed in the present section.

### 5.1. Rheologic Properties

The thermoplastic characteristics of formulations to produce filaments and then dosage forms change dramatically with modifications of raw materials, particularly on what concerns the rheological properties [16,126], very much dependent on the drug to polymer ratio [127]. The formulation may ultimately need the inclusion of a plasticizer to be printable [3]. Viscosity is the main rheological property of materials and is dependent on temperature [123]. Since both HME and FDM are thermal processes, heat during extrusion and printing, results in decreased viscosity of materials. An optimal formulation must present suitable viscoelastic properties and also unique yield stress under shear [128]. Polymers are macromolecules, which exhibit non-Newtonian behavior, usually presenting shear thinning (pseudo-plasticity) under flow. Moreover, they are viscoelastic in nature, i.e., presenting a combination of properties of both viscous liquids and elastic solids, depending on the time scale of the deformation imposed on them, very much conditioning their behavior toward the processing of the melt [123]. Often, polymer blends must be considered to improve the thermal and physical properties of the filaments, but also increasing the complexity of the system [4]. The addition of a drug, plasticizers, fillers, and other excipients to the formulation affects the viscosity of the polymer, or polymers, with the need for constant adjustment of the manufacturing parameters (e.g., temperature and extrusion/printing rate) [67,120,129]. Knowledge of the rheological properties of the polymer per se is not sufficient to anticipate the behavior of blends, since each component and the respective fraction modify the rheology of the polymer.

The viscoelasticity of a material, due to the plasticity that is time dependent, implies that the use of different shear stresses and shear rates (shear, extensional deformation, deformation rate) impact differently on the material. On the other hand, due to the elasticity of the same material, it tends to recover the original properties over time. Consequently, the time span between the preparation of the filament and its use in the printer and the time lapse between the preparation of the dosage form and its use by the patient may also be critical. The viscoelasticity may be expressed by the storage modulus (elasticity) and the loss modulus of viscosity (plasticity). Knowledge of both parameters allows a better prediction of the materials’ behavior.

The rheological properties of the formulations impact on the nozzle diameter, feed rate, and pressure drop. Therefore, the choice of the materials is paramount, and viscosity changes with temperature and shear stress while extruding or printing have to be taken into account. As such, two formulations with different viscosities may present similar printing abilities depending on the shear stress and shear rate imposed on them. Likewise, different printing rates may be required for formulations of the same polymer(s). Although the ability to print is the best assessment of viscosity, it is important that formulators identify the viscosity of formulations beforehand to understand and anticipate the performance of materials while printing. To that end, viscosity measurements are preferably performed by capillary rheometry, as compared to rotational rheometry [130].

As mentioned before, the viscosity of a mixture of materials must be low enough to enable flow and allow the extrusion of the filament or the printing of the dosage form [131] but not too low to prevent flow as a liquid. This means that the materials in a formulation should recover quickly to their original state, after removal of heat and shear to enable the construction of the dosage forms [132].

The yield stress and thixotropy must also be understood to anticipate the behavior of materials while printing. This will help definition of both temperature and rate of printing. Once the process parameters are adjusted, materials present the most appropriate viscosity for printing.

### 5.2. Mechanical Properties

Knowledge of the mechanical properties of HME filaments is paramount to anticipate their processing ability in HME and FDM 3D printing [133]. For each specific formulation, these properties must fall within narrow ranges. Stiffness and brittleness are the most important mechanical properties of a feedstock filament and should be assessed to evaluate its suitability for FDM 3D printing, together with elasticity (described by the Young’s modulus of elasticity) and plasticity, a time-dependent property. Stiffness reflects the deformation of a filament or dosage form (e.g., tablet) when a force is applied to the sample. The adequate stiffness of a filament should prevent spooling and allow the material to exit from the printer head. Stiffness should be considered for the entire formulation and not only for the single polymer; the addition of drug(s) (and indeed other components) is likely to affect this property. Brittleness reflects the ability of a ductile structure of a solid sample to deform plastically before it fractures. A brittle material has low ductility [134] and breaks, without showing significant plastic deformation, when submitted to a loading force. In 3D printing, low brittleness is required to prevent fracture of the filament while heated inside the printer’s head and to withstand traction during feeding. To decrease the brittleness of a material, plasticizers may be used in the filament’s composition, or resort to polymer blends. It is therefore critical to balance stiffness and brittleness of a filament to enable printing [72]. Dynamic mechanical analysis (DMA), flexure tests (three or four-point bending test), tensile test, or torsional strength are common techniques to determine these properties [80]. As previously discussed, filaments should, neither be excessively stiff because they will not be properly bent onto spools, nor excessively brittle to allow filaments to be properly loaded into the printer head without breaking during the process of printing, which ultimately lead to the blockage of the nozzle [41]. Blockage of the printer’s head is problematic because residues of filaments retained in the printing head can result in cross contamination (thus compromising the quality of the final dosage forms) and make cleaning of the equipment more difficult, requiring disassembling of part of or the entire apparatus.

Friability and hardness are standard official methods, which reflect the mechanical properties of dosage forms, such as tablets. However, due to the nature of the technologies (extrusion and printing), tablets usually present low friability and hardness; on the other hand, tensile strength is difficult to measure due to plastic deformation under an applied force.

### 5.3. Thermal Properties and Other Characterization at Molecular Level Techniques

A solid material can be amorphous, crystalline, or a combination of both states, with islands of crystalline arrangements, randomly distributed in an amorphous continuum. Variations in the thermodynamic state of a material lead to variations in its physical properties (e.g., viscosity) that impact processability. Therefore, the identification of the transitions of phase can anticipate the performance of the materials when extruded or printed [135]. In this respect, the specific heat capacity, thermal conductivity, density, or the existence of glass transition temperature(s) (Tg) or a melting point (Tm) of the polymers must be well-known. These are characteristics that directly impact fundamental properties of the materials—namely on the mobility of molecules, which in turn influence viscosity, brittleness, stiffness, and adhesion [136]. The Tg of a material, for instance, is relevant for a number of reasons. Below Tg, the viscosity is high, hindering flow; above Tg, with the increased mobility of molecules, their adhesion also increases, reducing viscosity and promoting flow. Tg is, therefore, a critical process parameter, which must be taken into account [137] for successful printing.

Thermal analysis of materials can be performed by a group of techniques in which a physical property (e.g., changes in energy, temperature, mass) of the material is measured, as a function of temperature, by subjecting the material to a controlled temperature program [138]. These techniques are normally used to monitor endothermic processes (e.g., glass transition, melting, solid–solid phase transition) and exothermic processes (e.g., crystallization, chemical degradation). Commonly used thermal analysis methods include differential scanning calorimetry (DSC), modulated differential scanning calorimetry (MDSC), thermogravimetric analysis (TGA), and isothermal microcalorimetry [138].

These techniques should be associated with the HME-coupled FDM 3D-printing technologies since thermal properties of the filament impact on the definition of the processing conditions, which ultimately may not accommodate the filament’s requirements. The temperature required to soften the filament without promoting the degradation of the materials (and the incorporated drug) and the environmental controlled-temperature around the building plate needed for the adequate bonding between the layers and cooling of the printed geometry need to be carefully selected. The thermal analysis of materials also plays an important role on drug–polymer solubility, anticipating their miscibility. Similarly, thermal analysis may help to assess the effect of moisture content on filament plasticity, another importance feature to control for adequate printing, as discussed before.

Thermal analysis of raw materials, filaments, and dosage forms is thus mandatory. Amongst the different techniques available, DSC is the most used [13], comparing the heat absorption by a sample with a standard. Likewise, TGA has been also used as a powerful technique for the measurement of thermal stability of materials, including API and polymers, measuring the weight change as a function of time and temperature, thereby providing information about the chemical stability of the material [57,74] and the compatibility of different materials in a solid dispersion mixture [139].

Polarized light microscopy (PLM) combines the use of heat with the ability of direct observation with an optical microscope, enabling the visualization of state transitions of materials. The possibility of attaching a video camera to the microscope allows the observation and recording of such transitions (soften and melting of solid samples on heating and recrystallization of molten samples on cooling). X-ray powder diffractometry (XRPD) is another important tool for studying both amorphous and semi-crystalline polymers and drugs. It can be used to analyze many features of the microstructure of the material, including lattice parameters, presence of imperfections, crystallographic orientations and degree of crystallinity [138]. In this respect, XRPD is used to confirm the production of ASD formed by the dispersion of a crystalline drug in a polymer upon HME filament production.

The ability of the printed layer to adhere to the previous one is paramount for the production of the desired dosage form. Several techniques can be considered to assess the surface energy of materials impacting on their interfacial adhesion. The latter reflects the intermolecular interaction between layers of printed material, directly related to the construction of the dosage form. The adhesion can be determined by surface tension measuring techniques or by torsion tests on the printed specimen [140] Adhesion is also related to the mechanical properties, namely brittleness [141,142].

Different spectroscopic techniques have also been considered to analyze the filaments and the dosage forms produced, such as tablets. For instance, the content of the drug can be quantified by ultraviolet/visible spectroscopy, either using a UV/visible spectrophotometer, or a detector coupled to a high performance chromatographer, provided the drug is released from the filament or dosage form and dissolves in a suitable solvent. Fourier-transform infrared spectroscopy (FTIR) can be used to determine whether two polymers, or a polymer and a drug, have formed bonds between each other anticipating properties relevant for printing, such as adhesion or viscosity. Typical features in the FTIR spectra suggest the presence, or absence, of bonds between the molecules of individual materials. Near-infrared spectroscopy (NIR) has also been considered to quantify the drug in the filament or tablets [143]. On the other hand, the use of Raman spectroscopy has been reported to provide evidence on the distribution of a drug within the filament or tablet [144]. Solid-state nuclear magnetic resonance (ssNMR) can also be considered to further elucidate the interactions between atoms of different molecules helping the identification of changes between formulation components.

### 5.4. In Vitro and In Vivo Characterization

Several researchers considered dissolution and disintegration tests to characterize the release profile of drugs embedded in 3D-printed dosage forms and estimate their suitability for the intended purpose (e.g., immediate or sustained/controlled/delayed release of the drug) [25]. Disintegration and dissolution [8] tests are performed in media with different complexities, ranging from water to biorelevant dissolution media (e.g., 0.1 M HCl to ascertain gastro-resistance).

Franz cells [16] can be used to quantify the permeation of a drug released from a printed tablet into a collecting compartment, using synthetic membranes or ex vivo tissues to test permeation. In vivo tests have been performed in animals (rats, dogs) [35] and have more recently moved into humans (clinical trials).

### 5.5. Other Properties

Dimensions and geometric features of both filament and 3D-printed specimens can be determined using a digital caliper, a laser micrometer, or ultrasonic thickness gauges [145]. Additionally, microscopic techniques (optical or scanning electronic microscopies) have been used. Accurate and precise diameters are required for the filaments while the length should be at least 20 cm long to feed the printer’s gears [83]. For a nozzle with 0.20 mm diameter, the filament often has a diameter of 1.75 ± 0.05 mm to pass through the driving gear and into the heater, although some printers allow diameters ranging between 1.75–3.00 ± 0.05 mm [83]. For many printers (e.g., MakerBot^®^ 3D printer), these are the diameters required for the filament [41]. Often, extrudates need to be pulled through a gauge to maintain the desired and uniform diameter [25,68], enabling the discharge of portions with a non-acceptable diameter [25]. To minimize variations of diameter, some authors have used lubricants (e.g., magnesium stearate), which, by reducing the friction between the extruding material and the extruder screw, enable the filament to exit the nozzle more steadily [5,41,47,56,58]. Uniformity of diameter is essential to guarantee consistent printing, since irregular dimensions may result in inconsistencies and print failure [41].

Besides filaments, 3D-printed dosage forms must have suitable dimensions and weight uniformity. Weight variation can be the result of inadequate rheological properties of the melt. In fact, while low viscosity results in smaller weight variation, higher viscosity is responsible for the production of tablets with unacceptable deviation [86].

Surface morphology of the 3D-printed dosage forms was also often evaluated by researchers using scanning electron microscopy (SEM) and transmission electron microscopy (TEM). SEM images show the filament surface and the structure of the fabricated 3D dosage form, revealing their construction from overlaid layers of filaments with a specific height. Several polymeric formulations composed of PVP [58] and cellulose-derivatives [4,86] revealed a smooth surface and tight structures of both filament and 3D structures. The existence of pores on the surface or surface imperfections should be monitored, as well as the structure of the tablets. For this, cross-sections can depict imperfections when observed by optical, electronic (SEM), or atomic force (ATM) microscopies [146]. These results may be complemented by measurement of the density (pycnometry) and/or by X-ray micro computed tomography [49].

## 6. Final Remarks

The manufacture of customized solid dosage forms has been made possible by 3D-printing technologies. In this work, FDM (the most used 3D-printing technology) in combination with HME (an already mature technology) was reviewed, from the perspective of the core raw materials (thermosoftening polymers) in use.

As discussed in the manuscript, not all polymers that are amenable to FDM are suitable for pharmaceutical applications. On the other hand, thermoplastic polymers traditionally used in HME may not perform well in FDM, which requires higher processing temperatures. Moreover, incorporation of the drug(s) or additives in the polymer is likely to change its physical and thermal properties and, therefore, extrudability and printability. As a consequence, processing parameters or formulation have to be adjusted to meet the requirements of both techniques.

This paper reviews the many strategies reported in the literature as a means to make a scientifically sound and systematic approach to polymer selection for additive manufacturing. Relevant aspects, such as the target population, the desired drug(s) release profile, the need to address drug solubility issues, taste masking or incompatibilities, and simplifying therapy management or therapeutic schedules, are also considered. The design of multi-layered or multi-compartmented dosage forms (e.g., comprising a core and a shell with different drugs or doses) are examples of the many strategies made possible by FDM.

FDM is mostly a process of extrusion and, because of that, fundamentals of this technology must be continuously recalled. For instance, the role of the polymer in formulations is core to the technology and the final product, and currently, its fraction in formulations must be above 50%. In fact, the choice of polymer and its fraction in the dosage form or particle size ultimately govern drug release. However, dosage form-related parameters such as size, geometry, and infill, which are easily changed and impact drug content and release profile, are also paramount. Polymer blends are a further possibility to adjust the properties for correct extrusion and printing, as well as to attain the desired drug release rate/profile. In addition, drug(s) and water may act as plasticizers, impacting on the brittleness/flexibility of filaments and, thus, on the quality of printing; optimal time after HME and storage conditions should also be observed.

The list of pharmaceutical-grade polymers available covers the functionality required for a matrix, but their use is often restricted by stability issues, since both technologies involve heating. In many instances, there has to be a compromise between the temperature required for processing and the chemical stability of materials (namely of the polymer and drugs). In the end, confirmation of the chemical and physical properties of the dosage form printed, as well as the pharmacopoeia standards, must be considered. The main characterization techniques used to ascertain quality in-process and of the final product are covered as well.

In general terms, the cellulosic-derivatives filaments (HPMC, HPC, HPMCAS, and EC) were prepared at extrusion temperatures ranging from 120–200 °C and printing temperatures ranging from 160–200 °C. On the other hand, PVA filaments were prepared at higher extrusion and printing temperatures (160–190 °C and 150–220 °C, respectively). Formulations with PEG/PEO, Soluplus^®^ (polyvinyl caprolactam-polyvinyl acetate-polyethylene glycol graft copolymer (PCL-PVAc-PEG)) and Eudragit^®^ RL exhibited lower extrusion temperatures (80–130°C) and printing temperatures below 200 °C for all formulations. The majority of the existing literature on pharmaceutical applications of FDM reports printing temperatures in the range of 150–230 °C, primarily using PVA, PLA, HPC, and other cellulose derivatives, and various grades of Eudragit polymers (RL, RS, and E), since these polymers extrude and print well. However, except for Eudragit E and HPC, many of these are not adequate for immediate release of the drug. A few studies investigate the production of such 3D-printed dosage forms, using PVP, PEO, and PCLa, which print at relatively lower temperatures, in the range of 100–130 °C.

Bearing in mind these considerations, the formulator can benefit from the polymers characteristics and performance, and design individualized dosage forms (e.g., immediate release of a drug with Kollicoat^®^ IR and PEO; swellable/erodible system with HPC, HPMC, PVA, and Soluplus^®^; slowly permeable/insoluble with EC and Eudragit^®^ RL; and gastro-resistant with Eudragit^®^ L and HPMCAS).

**Figure 13:**
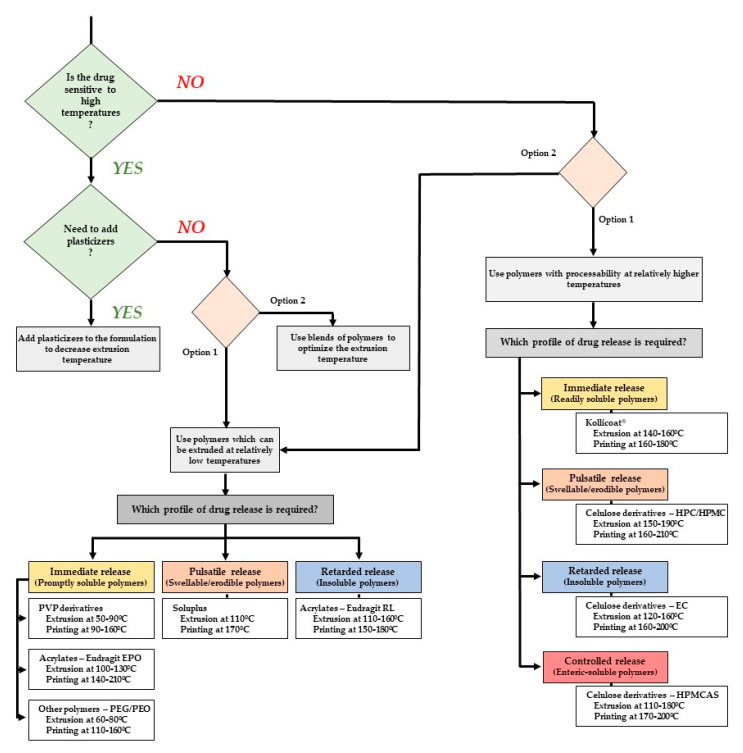
Decision tree for the choice of a polymer for HME coupled to FDM, considering drug thermal sensitivity, impact of thermal properties of the polymer on extrudability/printability, and desired drug release profile.

Aiming at expediting the formulation process, a decision tree diagram (Figure 13) has been included in this section. The selection of the matrix forming polymer(s) with the desired properties should be more straightforward, thus reducing iterations and guaranteeing drug stability at the same time.

As industry is investing in bringing new polymers to the market specifically designed to meet the needs of HME-coupled FDM, it is likely that the future will provide the exact tools to fulfil the dream of personalized medicine.

## Figures and Tables

**Table 1:** Most common drugs used in FDM 3D printing and their characteristics.

Drug	BCS Class ^1^	Therapeutic Class	Characteristics			Works Reporting HME Coupled to FDM
			Molecular Weight (g/mol)	Melting Point (°C)	Solubility at 37 °C (mg/mL)	
4-Aminosalicylic acid	II	Antitubercular agent	153.14	150	2.0	[47]
5-Aminosalicylic acid	IV	Anti-inflammatory	153.135	275	0.1	[4,29,45]
Acetaminophen	I	Anti-inflammatory	151.16	170	218.0	[4,42,48,49,50]
Amlodipine besylate	II	Calcium channel blocker	408.88	198	0.207	[51]
Amoxicillin	IV	Antibiotic	365.40	194	0.958	[52]
Aripiprazole	IV	Atypical antipsychotic	448.38	140	0.00777	[53]
Ascorbic acid	I	Vitamin/Antioxidant	176.12	191	330.0	[54]
Baclofen	III	Gamma-aminobutyric acid agonist	213.66	207	0.712	[55]
Bicalutamide	II	Non-steroidal anti-androgen	430.37	192	0.00928	[53]
Budesonide	II	Corticosteroid	430.50	226	0.0457	[4,50,56]
Caffeine	II	Bronchodilator or vasodilator	194.19	234	11.0	[50]
Captopril	I	Angiotensin-converting-enzyme inhibitor	217.29	427	160.0	[29]
Carvedilol	II	Antihypertensive	442.90	114.5	0.00444	[57]
Ciprofloxacin	IV	Antibiotic	318.10	581.8	1.35	[43]
Diclofenac sodium	II	Anti-inflammatory	430.50	285	0.00482	[56]
Domperidone	II	Antiemetic	425.91	242	0.986	[13]
Dronedarone hydrochloride	II	Antiarrhythmic	556.76	140	0.00201	[54]
Dypyridamole	II	Antiplatelet drug	504.63	163	0.922	[58]
Felodipine	II	Antihypertensive	384.26	471	19.7	[42]
Glimepiride	II	Antidiabetic	490.62	207	0.0384	[59]
Glipizide	II	Antidiabetic	445.53	200	0.0164	[60]
Haloperidol	II	Antipsychotic	375.90	148	0.00446	[61,62]
Hydrochlorothiazide	II	Diuretic	297.74	267	2.24	[59]
Ibuprofen	II	Anti-inflammatory	206.29	319	0.021	[42,63]
Indapamide	II	Antihypertensive	365.84	161	0.0342	[51]
Indomethacin	II	Anti-inflammatory	357.80	162	0.937	[64,65]
Itraconazole	II	Antifungal agent	705.64	166	0.00964	[66]
Lisinopril dihydrate	III	Antihypertensive	405.49	148	0.216	[51]
Metformin	II	Antidiabetic	129.16	172	1.38	[59]
Nitrofurantoin	II	Antibiotic	238.16	225	0.415	[67]
Pantoprazole	II	Proton pump inhibitor	383.37	155	0.431	[68]
Prednisolone	I	Corticosteroid	360.44	230	0.24	[7,29]
Quinine	I	Antimalarial	324.42	57	0.334	[69]
Ramipril	II	Antihypertensive	416.50	109	0.035	[47]
Rosuvastatin calcium	II	Antihyperlipidemic	481.54	155	0.0886	[51]
Theophylline	I	Bronchodilator	180.16	270	5.5	[7,26,29,56,58,70,71,72,73]
Warfarin	I	Anticoagulant	308.32	161	0.0472	[74]

^1^ BCS Class: I–High solubility, high permeability; II–High permeability, low solubility; III–Low permeability, high solubility; IV–Low solubility, low permeability.

**Table 2:** Characteristics and thermal properties of the polymers used in hot-melt extrusion (HME)-coupled to FDM.

Chemical Name of the Polymer	Trade Name	FDA Approved	Biodegradable Polymer	Thermal Properties ^1^			References
				Tm (°C)	Tg (°C)	Degradation Temperature (°C)	
Polyvinyl alcohol (PVA)	---	Yes	Yes	---	85	---	[3,27,77,82]
Poly(vinylpyrrolidone) (PVP)							
(MW 7000–11,000)	Kollidon^®^ 17 PF	Yes	Yes	---	140	217	[56,58,68]
(MW 2000–3000)	Kollidon^®^ 12 PF	Yes	Yes	---	72	196	
Poly(vinylpyrrolidone)/vinyl acetate (PVP/VA) (MW 45,000–70,000)	Kollidon^®^ VA64	Yes	Yes	---	105	270	[78]
Poly(vinyl caprolactam-covinylacetate-ethylene glycol (MW 90,000–140,000)	Soluplus^®^	Yes	Yes	---	72	278	[4,42]
Hydroxypropyl cellulose (HPC) (MW 95,000)	Klucel^®^ LF	Yes	Yes	---	111	227	[13,36,83]
Hydroxypropylmethyl cellulose (HPMC)							[84,85] [76,82]
(MW 25,000)	Methocel™ K100LV	Yes	Yes	168	147	259	
(MW 150,000)	Methocel™ K100M	Yes	Yes	173	96	259	
Hydroxypropyl methyl cellulose acetate succinate (HPMCAS)	Affinisol™	Yes	Yes	---	115	<250	[25,35,65,86,87]
Poly ethylene oxide (PEO)	---	Yes	Yes	100	−67	340	[88]
Poly(butyl methacrylate-*co*-(2-demethylamino ethyl) methacrylate-*co*-methyl methacrylate) 1:2:1	Eudragit E PO^®^	Yes	Yes	189–193	52	250	[29,42]
Poly(ethyl acrylate-*co*-methyl methacrylate-*co*-trimethylammonio ethyl methacrylate chloride) 1:2:0.2	Eudragit RL^®^	Yes	Yes	---	63	166	[5,18,25]
Poly(methacrylic acid-*co*-methyl methacrylate) 1:1	Eudragit L^®^	Yes	Yes	---	111	176	[77]
Poly(ethyl acrylate-*co*-methyl methacrylate-*co*-trimethylammonioethylmethacrylate chloride) 1:2:0.1	Eudragit RS^®^	Yes	Yes	---	64	170	[77]
Poly(e-caprolactone) (PCLa)	---	Yes	Yes	60	---	310	[5]
Ethylene vinyl acetate (EVA)	---	Yes	Yes	70	91	300	[64]
Ethylcellulose							[77]
(4 cPs)	Ethocel^®^ 4P			168	128	200	
(7 cPs)	Ethocel^®^ 7P	Yes	Yes	168	128	205	
(10 cPs)	Ethocel^®^ 10P			172	132	205	
Polyvinyl alcohol;polyethylene glycol graft copolymer (PVA:PEG)	Kollicoat^®^ IR	Yes	Yes	---	208	200	[77]

^1^ Note that the crystalline region is characterized by T_m_ while T_g_ is a property related to the amorphous region of the polymers; thus, amorphous polymers do not have T_m_ [78]. T_m_–Melting point; T_g_–Glass transition temperature.

**Table 3:** Pharmaceutical grade polymers used to prepare filaments (HME) for FDM 3D printing. The composition (% *w/w*) of the filaments (polymer, drug and adjuvants) and polymer manufacturer are specified.

Main Matrix-Former Polymer	Polymer (% *w*/*w*)	Drug (% *w/w*)	Other Components			Polymer Trade Name (Manufacturer)	Ref.
			Plasticizer (% *w/w*)	Lubricant/Filler (% *w/w*)	Other (% *w/w*)		
ALCOHOL-DERIVED POLYMERS							
Polyvinyl alcohol (PVA)	65–90	Ciprofloxacin (10–35)	Dibutyl sebacate (0.20)	---	---	Natural PVA (Ultimaker, Utrecht, The Netherlands)	[43]
	95	Budesonide (5)	---	---	---	Commercial filament (Makerbot Inc., New York, NY, USA)	[89]
	100	Fluorescein	---	---	---	Commercial filament (Makerbot Inc.)	[27]
	100	Curcumin	---	---	---	Commercial filament (---)	[17]
	56–70	Lisinopril dihyd. (20) Amlodipine besylate (10) Indapamide (5) Rosuvastatin Calcium (20)	Sorbitol (23–30) Titanium dioxide (1)	---	---	PVA (Parteck MXP)	[51]
	90, 95	Acetaminophen or Caffeine (5,10)	---	---	---	PVA (Makerbot Inc.)	[50]
	80	Baclofen (10)	Sorbitol (10)	---	---	PVA (Parteck MXP)	[55]
	84	Hydrochlorotiazide (6)	Mannitol (10)	---	---	Mowiol 4–88 (Sigma Aldrich, St. Louis, MO, USA)	[40]
	97.8, 95.2	Glipizide (2.2, 4.8)	---	---	---	---	[60]
	67.5	Felodipine (10)	Tween 80 (22.5)	---	---	PVA MW 18,000–25,000 (Colorcon, Lower Salford Township, PA, USA)	[42]
	90, 80, 70	Carvedilol (10 or 20) Haloperidol (10 or 20)	Sorbitol (10)	---	---	PVA (Parteck MXP)	[62]
	98.8	Aripiprazole (1.2)	---	---	---	Poval 4–88 (Kuraray, Kurashiki, Japan)	[90]
	63 or 73.8	Acetaminophen (20)	---	---	Disintegrant: Sodium starch glycolate (7) Croscarmellose (7)	PVA MW 89,000–98,000 (Sigma Aldrich)	[91]
POLY-(VINYLPYRROLIDONE) AND RELATED POLYMERS							
Poly-(vinylpyrrolidone) (PVP)	50	Theophylline (10)	Triethyl citrate (12.5)	Talc (27.5)	---	PVP MW 40,000 (Sigma-Aldrich)	[58]
	50	Dipyridamole (10)	Triethyl citrate (12.5)	Talc (27.5)	---		
	32.5	Ramipril (3)	PEG 1500 (20) Mannitol (10)	Magnesium Carbonate (2)	Polymer: Kollidon^®^ VA64 (32.5)	Kollidon^®^ 12PF (BASF, Ludwigshafen, Germany)	[47]
	45–75	Pantoprazole (10–30)	Triethyl citrate (15–25)	---	---	PVP K12 (Carl Roth, Karlsruhe, Germany)	[68]
	50	Theophylline (10)	Triethyl citrate (12.5)	Talc (27.5)	---	PVP MW 40,000 (Sigma-Aldrich)	[56]
		Theophylline (10)	Triethyl citrate (12.5)	Tribasic Phosphate Sodium (27.5)	---		
		Budesonide (2.3)	Triethyl citrate (12.5)	Talc (35.2)	---		
	45.5	Diclofenac sodium (20)	Triethyl citrate (17.5)	Talc (17)	---		
Poly(vinylpyrrolidone)/vinyl acetate (PVP/VA)	65	4-ASA or Ramipril (3)	PEG 1500 (20) Mannitol (10)	Magnesium Carbonate (2)	---	Kollidon^®^ VA64 (BASF)	[47]
	39	Ramipril (3)	PEG 1500 (20) Mannitol (10)	Magnesium Carbonate (2)	Polymer: Kollidon^®^ 12PF (26)		
	32.5	Ramipril (3)	PEG 1500 (20) Mannitol (10)	Magnesium Carbonate (2)	Polymer: Kollidon^®^ 12PF (32.5)		
	65	Pantoprazole (10)	Triethyl citrate (25)	---	---	Kollidon^®^ VA64 (BASF)	[68]
CELLULOSE-DERIVED POLYMERS							
Ethylcellulose (EC)	90	---	Triethyl citrate (10)	---	---	Ethocel™ (Dow, Midland, MI, USA)	[25]
	100	---	---	---	---	Aqualon™ EC N14 (Ashland Inc., Wilmington, NC, USA)	[86]
	70	Acetaminophen (30)	---	---	---		
	70	Acetaminophen (30)	---	---	---	Aqualon™ (Ashland Inc.)	[4]
	35	Acetaminophen (30)	---	---	Polymer: HPMC (35) or Soluplus^®^ (35)		
	50	Acetaminophen (30)	---	---	Polymer: Eudragit^®^ L100 (15) Disintegrant: Kollidon^®^ CL-F (5)		
	60	Ibuprofen (20)	---	---	Release Modifiers: Sodium Alginate (20) or PVA (20) or Xanthan Gum (20)	EC Std.10 No.PD416124 (Colorcon)	[63]
	50–70	Ibuprofen (20)	---	---	Release Modifier: HPMC (10–30)		
	57–63	Ibuprofen (16–24)	---	---	Release modifier: HPMC (19–21)		
	75	---	Methylparaben (20)	Magnesium Stearate (5)	---	Aqualon N7 (Ashland Inc.)	[35]
	60	Quinine (5)	Triacetin (35%)	---	---	---	[69]
Hydroxypropyl cellulose (HPC)	100	---	---	---	---	Klucel^®^ LF (Ashland Inc.)	[25]
	100	---	---	---	---	Klucel™ HPC EF (Ashland Inc.) Klucel™ HPC HF (Ashland Inc.)	[86]
	70	Acetaminophen (30)	---	---	---		
	100	---	---	---	---		
	70	Acetaminophen (30)	---	---	---		
	70	Acetaminophen (30)	---	---	---	Klucel™ HPC EF (Ashland Inc.)Klucel™ HPC LF (Ashland Inc.)	[4]
	35	Acetaminophen (30)	---	---	Polymer: HPMC (35) or EC N14 (35)		
	19.5	Acetaminophen (30)	---	---	Polymer: HPMC E5 (45.5) Disintegrant: Kollidon^®^ CL-F (5)		
	73.75	---	Mannitol (21.25)	Magnesium Stearate (5)	Colorants: Candurin Orange Amber (1) Candurin Gold Sheen (1) Food Colouring (5)	Klucel^®^ ELF (Ashland Inc.)	[91]
	46	Theophylline (50)	Triacetin (4)	---	---	HPLC SSL (Nisso Chemical Europe, Düsseldorf, Germany)	[26]
	45	Theophylline (50)	Triacetin (5)	---	---	HPLC SSL (Nisso Chemical Europe)	[36]
	45	Theophylline (45)	Triacetin (5)	---	Disintegrant (5): Croscarmellose Sodium (Ac-Di-Sol^®^) or Sodium Starch Glycolate (Primojel^®^ and Explotab^®^) or Crospovidone (Polyplasdone™ XL-10)		
	90–100	Acetaminophen (trace amounts)	PEG (0–10)	---	---	Klucel^®^ LF (Ashland Inc.)	[83]
	90	Domperidone (10)	---	---	---	Klucel^TM^ EXF (Ashland Inc.)	[13]
	80	Domperidone (10)	---	---	Opalescent agent: Barium Sulphate (10)		
	65	Itraconazole (20)	---	---	Solubility enhancer: PVP (15)	HPC-SL (Nippon Soda Co. Ltd., Tokyo, Japan)	[66]
	100	---	---	---	---	Klucel LF (Ashland Inc.)	[71]
	99.5	---	---	---	Polymer: EC (0.5)		
	73.75	---	Mannitol (21.25)	Magnesium Stearate (5)	---	Klucel^®^ EF (Ashland Inc.)	[35]
Hydroxypropyl methylcellulose (HPMC)	95	---	PEG 400 (5)	---	---	Affinisol™15cP (Dow)	[25]
	100	---	---	---	---	Benecel™ HPMC E5 (Ashland Inc.) Benecel™ HPMC K100M (Ashland Inc.)	[86]
	70	Acetaminophen (30)	---	---	---		
	100	---	---	---	---		
	70	Acetaminophen (30)	---	---	---		
	70	Acetaminophen (30)	---	---	---	Benecel™ HPMC E5 (Ashland Inc.)	[4]
	35	Acetaminophen (30)	---	---	Polymer: (35) EC N14 or HPC EF or HPC LF or Soluplus^®^ or Eudragit^®^ L100		
	45.5	Acetaminophen (30)	---	---	Polymer: (19.5) EC N14 or HPC EF or HPC LFor Soluplus^®^ or Eudragit^®^ L100		
	50	Acetaminophen (30)	---	---	Polymer: (19.5) EC N14 or HPC EF or HPC LF or Soluplus^®^ or Eudragit^®^ L100		
	70	Acetaminophen (10)	---	---	Solubilizer: Soluplus^®^ (20)	Benecel™ HPMC E5 (Ashland Inc.)	[48]
	60	Carvedilol (20)	Kolliphor TPGS (5)	---	Polymer: udragit PO (15)	Affinisol™ 15cP (Dow)	[57]
	95	Acetaminophen (trace amounts)	PEG 400 (5)	---	---	Affinisol™ 15cP (Dow)	[34]
	70–90	Haloperidol (10–30)	---	---	---	Affinisol™ 15cP (Dow)	[61]
	20, 40	Nitrofurantoin (5)	---	---	Polymer: PLA (55, 75)	Affinisol™ 15cP (Dow)	[67]
Hydroxypropyl methylcellulose acetate succinate (HPMCAS)	95	---	PEG 8000 (5)	---	---	AQUOT^®^-LG (Shin-Etsu, Tokyo, Japan)	[25]
	100	---	---	---	---	AquaSolve™ HPMCAS LG (Ashland Inc.) AquaSolve™ HPMCAS HG (Ashland Inc.)	[86]
	70	Acetaminophen (30)	---	---	---		
	100	---	---	---	---		
	70	Acetaminophen (30)	---	---	---		
	80	Acetaminophen (trace amounts)	PEG 8000 (20)	---	---	AQUOT^®^-LG (Shin-Etsu)	[34]
	40,75	Acetaminophen (5,50)	Methylparaben (5,15)	Magnesium Stearate (5)	---	AQUOT^®^-HG (Shin-Etsu)	[49]
	70	---	Methylparaben (15)	Magnesium Stearate (5) Talc (10)	---	Aquasolve™-LG (Ashland Inc.)	[35]
	60	Indomethacin (20)	PEG 6000 (20)	---	---	AQUOT^®^-AS-MF (Shin-Etsu)	[65]
ACRYLATES							
Poly(butyl methacrylate-*co*-(2-demethylaminoeethyl) methacrylate-*co*-methyl methacrylate) 1:2:1	50–55.56	Felodipine (10)	PEG 4000 (15)	---	Polymer: PEO (15) Solubility Enhancer: Tween 80 (10)	Eudragit^®^ E PO (Evonik Industries, Essen, Germany)	[42]
	100	---	---	---	---	Eudragit^®^ E PO (Evonik Industries)	[44]
	55.5	---	Tween 80 (11.1)		Polymer: PEG or PEO (16.7)	Eudragit^®^ E PO (Evonik Industries)	[44]
	46	5-ASA, Captopril, Prednisolone or Theophylline (12.5)	Triethyl citrate (6.5)	---	Disintegrant: Tri-calcium phosphate (37.5)	Eudragit^®^ E PO (Evonik Industries)	[29]
	46.5	Warfarin (1)	Triethyl citrate (3)	---	Disintegrant: Tri-calcium Phosphate (49)	Eudragit^®^ E PO (Evonik Industries)	[74]
	46.5	Theophyline (50)	Triethyl citrate (3.5)	---	---	Eudragit^®^ E (Evonik Industries)	[26]
Poly(ethyl acrylate-*co*-methyl methacrylate-*co*-trimethylammonio ethyl methacrylate chloride) 1:2:0.2	59.6–69.6	Theophyline (30)	Stearic acid (3.5–7) or PEG 4000 (5–10)	Anhyd. colloidal silica (0.4)	Polymer: HPC (84.68)	Eudragit^®^ RL PO (Evonik Industries)	[72]
	62.6	Theophyline (30)	Stearic acid (7) PEG 4000 (10)	Anhyd. colloidal silica (0.4)	---	Eudragit^®^ RL PO (Evonik Industries)	[18]
	64	---	Triethyl citrate (6) Mannitol (20)	PEG 6000 (10) Microcrystalline cellulose (20)	---	Eudragit^®^ RL 100 (Evonik Industries)	[5]
	35–40	Glimepiride (2) Metformin (50)	PEG 400 (5–7) Triethyl citrate (5) Citric acid Monohydrate (10–15) Mannitol (15)	Calcium stearate (3)	Polymer: PLA (10)	Eudragit^®^ RL PO (Evonik Industries)	[59]
	45	Theophyline (50)	Triethyl citrate (5)	---	---	Eudragit^®^ RL 100 (Evonik Industries)	[26]
	85	---	Triethyl citrate (15)	---	---	Eudragit^®^ RL 100 (Evonik Industries)	[25]
Ethyl Prop-2-enoate; methyl 2-methylprop-2-enoate;Trimethyl-[2-(2-methylprop-2-enoyloxy)ethyl] azanium; chloride	95	Quinine (5)	---	---	---	Eudragit^®^ R (Evonik Industries)	[69]
Poly(methacrylic acid-*co*-methyl methacrylate) 1:1	80	---	Triethyl citrate (20)	---	---	Eudragit^®^ L (Evonik Industries)	[25]
Poly(ethyl acrylate-*co*-methyl methacrylate-*co*-trimethylammonio ethyl methacrylate chloride) 1:2:0.1	42.5	Theophyline (50)	Triethyl citrate (7.5)	---	---	Eudragit^®^ RS 100 (Evonik Industries)	[26]
OTHER POLYMERS							
Polyvinyl caprolactam-polyvinyl acetate-polyethylene glycol graft copolymer (PCL: PVA: PEG)	90	---	PEG 400 (10)	---	---	Soluplus^®^ (BASF)	[25]
	50	Felodipine (10)	Tween 80 + PEG 4000 + PEO WSR (15:10:15)	---	---	Soluplus^®^ (BASF)	[42]
	100	---	---	---	---	Soluplus^®^ (BASF)	[44]
	90	---	---	---	Polymer: PEG (10)	Soluplus^®^ (BASF)	[44]
	80	---	Tween 80 (20)	---	---	Soluplus^®^ (BASF)	[44]
Poly(e-caprolactone) (PCLa)	64	---	Mannitol (20)	PEG 6000 (10) Microcrystalline cellulose (20)	---	Capa^TM^ 6506 (Triiso, Cardiff, UK)	[5]
	95, 85, 70	Indomethacin (5, 15, 30)	---	---	---	Capa™ 6500 (Triiso)	[92]
	95	Quinine (5)	---	---	---	PCLa MW 14,000 (Sigma Aldrich)	[69]
Polyvinyl alcohol/polyethylene glycol graft copolymer (PVA: PEG)	88	---	Glycerol (12)	---	---	Kollicoat^®^ IR (BASF)	[25]
	88	Acetaminophen (trace amounts)	Glycerol (12)	---	---	Kollicoat^®^ IR (BASF)	[34]
	45	---	Methylparaben (20) Mannitol (20)	Magnesium Stearate (5) Talc (10)	---	Kollicoat^®^ IR (BASF)	[35]
	97.5	Bicalutamide (3.5)	---	---	---	Kollicoat^®^ IR (BASF)	[53]
	90	Haloperidol (10)	---	---	---	Kollicoat^®^ IR (BASF)	[61]
Polyethylene oxide (PEO)	58	Acetaminophen (20–40) Ibuprofen (20–40)	---	Sodium Lauril Sulfate (2)	Disintegrant: Starch (20) Sodium starch glycolate (7) Croscarmellose (2)	PEO 100K (Sigma-Aldrich)	[93]
	40–58	Ibuprofen (20–40)	---	Starch (18–20) Sodium Lauril Sulfate (1–2)	---	PEO 200K (Sigma-Aldrich)	
	35	Theophylline (30)	PEG 6K (35)	---	---	PEO 300K (Sigma-Aldrich)	[73]
	35	Theophylline (30)	PEG 6K (35)	---	---	PEO 600K (Sigma-Aldrich)	
Ethylene vinyl Acetate (EVA)	85, 95	Indomethacin (5, 15)	---	---	---	ATEVA 1070, 1075A, 1081G, 1241, 1641, 1821A, 1850A, 1880A, 2821A, 3325A (Celanese, Irving, TX, USA)	[64]

--- Information not available.

**Table 4:** Processing conditions and equipment used in HME of filaments and FDM 3D printing of dosage forms. The polymer used and the type of drug release obtained are given.

Matrix Polymer	HME Process				Extruder Model (Manufacturer)	FDM Process						Printer Model (Manufacturer)	Dosage Form Printed	Reference
	Extrusion Temperature (°C)	Screw Speed (rpm)	Torque (N/cm)	Nozzle Size (mm)		Printing Temperature (°C)	Extrusion Speed (mm/s)	Travelling Speed (mm/s)	Infill (%)	Nozzle Size (mm)	Layer Height (mm)			
ALCOHOL-DERIVED POLYMERS														
Polyvinyl alcohol (PVA)	---	---	---	---	---	220	90	150	0, 10, 25, 50 90, 100	---	0.20	Replicator 2 (MakerBot Ind., New York, NY, USA)	IM or ER tablets depending on infill	[27]
	---	---	---	---	---	140–250	20	---	0–100	---	0.80	FDM- 200W, (NinjaBot)	ER tablets	[17]
	160–175	30–60	8	0.30	Noztek Touch HT, (Noztek, Shoreham-by-Sea, UK)	195	8	150	100	0.4	0.30	Ultimaker 3 (Ultimaker)	CR tablets	[43]
	90	35	---	1.70	Haake™ MiniCTW (Thermo Fisher, Waltham, MA, USA)	150	---	---	100	---	0.50	MarketBot 2 (MakerBot Ind.)	MR tablets	[51]
	180	15	---	1.75	Noztek, (Noztek)	200	90	150	100	---	0.2	Replicator 2 (MakerBot Ind.)	MR capsules	[50]
	160	200	---	1.55	Twin Screw Extruder (Thermo Scientific)	170	---	---	30, 65, 100	---	---	--- (MakerBot Ind.)	IR minicaplets	[55]
	190	23	0.7	1.50	Filabot Original (Filabot, Inc., Boston, MA, USA)	90	70	90	100	---	0.2	Replicator 2 (MakerBot Ind.)	SR tablets	[40]
	---	---	---	---	---	25	0.0075	10	50	---	0.45	MAMII (Fochif Mech. Tech., Shanghai, China)	SR tablets	[60]
	100–130	23	8	1.75	Haake™ MiniCTW (Thermo Fisher)	150	---	---	100	0.4	0.20	Replicator 2 (MakerBot Ind.)	ER disks	[42]
	170–190	200	---	1.5	Twin Screw Extruder (Thermo Scientific)	210	50	150	60, 100	0.4	0.05	Replicator 2 (MakerBot Ind.)	IR tablets	[62]
	172	---	---	1.75	Noztek, (Noztek)	190	5	---	40	0.3	0.15	---	IR films	[90]
	130	100		1.6	Noztek, (Noztek)	190	90	150	100	0.4	0.2	Wanhao Duplicator 4 (Jinhua)	IR films	[93]
	POLY-(VINYLPYRROLIDONE) AND RELATED POLYMERS													
Poly-(vinylpyrrolidone) (PVP)	90	---	40	---	Haake™ MiniCTW (Thermo Fisher)	160 60	90	150	100	0.4	0.20	Replicator 2 (MakerBot Ind.)	IR tablets	[58]
	65	15	---	1.30	Noztec Pro Hot Melt (Noztec, Bentley, Australia)	90	90	150	100	---	0.20	Replicator 2 (MakerBot Ind.)	IR tablets	[47]
	47–60	4.5–7.5	---	2.80	---	79–87	60	---	50,90, 100	0.35	0.1	Multirap M420 (Multec GmbH, Illmensee, Germany)	IR tablets	[68]
	90	---	40	1.25	Haake™ MiniCTW (Thermo Fisher)	110 40	12	50	100	---	0.2	---	IR tablet core	[56]
Poly(vinylpyrrolidone)/vinyl acetate (PVP/VA)	70	15	---	1.30	Noztec Pro Hot Melt (Noztec)	90	90	150	100	---	0.20	Replicator 2 (MakerBot Ind.)	IR tablets	[47]
	65	15	---	1.30	Noztec Pro Hot Melt (Noztec)	90	90	150	100	---	0.20	Replicator 2 (MakerBot Ind.)	IR tablets	
	65	15	---	1.30	Noztec Pro Hot Melt (Noztec)	90	90	150	100	---	0.20	Replicator 2 (MakerBot Ind.)	IR tablets	
	54–56	4.5–7.5	---	2.80	---	85	60	---	100	0.35	0.1	Multirap M420 (Multec GmbH)	IR tablets	[68]
CELLULOSE-DERIVED POLYMERS														
Ethylcellulose(EC)	160	100	100	1.80	Haake™ MiniLab II (Thermo Fisher)	200	---	---	100	0.4	0.30	Replicator 2 (MakerBot Ind.)	ER disks	[25]
	150	50	72	---	(Thermo Fisher)	200 50	50	50	100	0.4	0.10	Prusa i3 (Prusa Research, Prague, Czech Republic)	ER tablets	[86]
	140–160	50	---	2.00	(Thermo Fisher)	200 50	50	50	100	0.4	0.10	Prusa i3 (Prusa Research)	ER tablets	[4]
	100–120	60	---	2.00	Haake™ MiniCTW, (Thermo Fisher)	170–186	7.5–75	100	15–25	0.4	0.10–0.30	JG Aurora A3 (JG Aurora, Shenzhen, China)	ER tablets	[63]
	120	15	---	1.75	Noztec Pro Hot Melt (Noztec)	160 0	3	25	100	---	0.10	Replicator 2 (MakerBot Ind.)	ER capsular devices	[35]
	59	0.94	---	3.00	---	145	144	40	100	0.35	0.10	Multirap M420 (Multec GmbH).	IR tablets	[69]
Hydroxypropyl cellulose (HPC)	165	80	40	1.80	Haake™ MiniLab II (Thermo Fisher)	180	---	---	100	0.4	0.30	Replicator 2 (MakerBot Ind.)	CR disks	[25]
	140–170	50	55–120	---	(Thermo Fisher)	200 50	50	50	100	0.4	0.10	Prusa i3 (Prusa Research)	ER tablets	[86]
	140–160	50	---	2.00	(Thermo Fisher)	200 50	50	50	100	0.4	0.10	Prusa i3 (Prusa Research)	ER tablets	[4]
	130	15	---	1.75	Noztec Pro Hot Melt (Noztec)	140	90	150	100	---	0.20	Replicator 2 (MakerBot Ind.)	CR caplets	[91]
	125–110	---	60	1.50	Haake™ MiniCTW (Thermo Fisher)	160 60	90	150	100	---	0.20	Replicator 2 (MakerBot Ind.)	IR tablets	[26]
	120	80	80	1.70	Haake™ MiniCTW (Thermo Fisher)	---	70	150	100	0.4	0.30	Replicator 2 (MakerBot Ind.)	IR tablets	[36]
	150–165	50–60	---	1.75	Haake™ MiniLab II (Thermo Fisher)	210	---	---	100	0.4	0.30	Replicator 2 (MakerBot Ind.)	PR capsular devices	[83]
	145–150	20–25	10–20	1.75	Haake™ MiniCTW (Thermo Fisher)	210 30	---	90	0, 10, 20, 30	---	0.20	Replicator 2 (MakerBot Ind.)	SR intra-gastric floating tablets	[13]
	135	35	---	1.75	Filabot Original EX2 (Filabot)	185 55	---	80	0	0.5	0.10	MF2200-D (Mutoh Industries)	SR floating tablets	[66]
	165	50	40–50	---	Process 11(Thermo Fisher)	190 60	50	50	50, 75, 100	0.4	0.10	Prusa i3 (Prusa Research)	SR floating tablets	[71]
	130	15	---	1.75	Noztec Pro Hot Melt (Noztec)	160 0	3	25	100	---	0.10	Replicator 2 (MakerBot Ind.)	CR tablets	[35]
Hydroxypropyl methylcellulose(HPMC)	160	70	70	1.80	Haake™ MiniLab II (Thermo Fisher)	200	---	---	100	0.4	0.30	Replicator 2 (MakerBot Ind.)	CR disks	[25]
	190	50	84	---	(Thermo Fisher)	200 50	50	50	100	0.4	0.10	Prusa i3 (Prusa Research)	CR tablets	[86]
	180	50	---	2.00	(Thermo Fisher)	200 50	50	50	100	0.4	0.10	Prusa i3 (Prusa Research)	CR tablets	[4]
	160	50	---	2.00	Haake™ MiniLab II (Thermo Fisher)	200 50	50	50	20–100	0.4	0.10	Prusa i3 (Prusa Research)	CR tablets	[48]
	110	50	---	---	Haake™ MiniLab II (Thermo Fisher)	180 RT	30	---	20	0.4	0.30	CraftBot Plus (Craftunique, Budapest, Hungary)	CR tablets	[57]
	160	70	70	1.80	Haake™ MiniLab II (Thermo Fisher)	200	---	---	100	0.3 0.4	0.10	Replicator 2 (MakerBot Ind.)	PR capsular devices	[34]
	150–170	200	---	1.55	Haake™ MiniCTW (Thermo Fisher)	210 RT	45	150	60–100	0.4	0.10	Replicator 2 (MakerBot Ind.)	IM tablets	[61]
Hydroxypropylmethylcellulose acetate succinate (HPMCAS)	180	100	100	1.80	Haake™ MiniLab II (Thermo Fisher)	200	---	---	100	0.4	0.30	Replicator 2 (MakerBot Ind.)	MR disks	[25]
	200	50	46–66	---	(Thermo Fisher)	200 50	50	50	100	0.4	0.10	Prusa i3 (Prusa Research)	MR tablets	[86]
	180	100	100	1.80	Haake™ MiniLab II (Thermo Fisher)	200	---	---	100	0.3 0.4	0.30	Replicator 2 (MakerBot Ind.)	PR capsular devices	[34]
	80–110	15	---	1.75	Noztec Pro Hot Melt (Noztec)	180–190	90	150	20,100	---	0.10	Replicator 2 (MakerBot Ind.)	MR tablets	[49]
	105	15	---	1.75	Noztec Pro Hot Melt (Noztec)	175 0	3	25	100	---	0.10	Replicator 2 (MakerBot Ind.)	CR tablets	[35]
	40–120	50	---	---	Eurolab 16 (Thermo Fisher)	165	25	----	7	---	0.15	HD2xR (Airwolf, Pigeon Forge, TN, USA)	MR Starmix^®^ structures	[65]
ACRYLATES														
Poly(butyl methacrylate-*co*-(2-demethylaminoeethyl) methacrylate-*co*-methyl methacrylate) 1:2:1 (Eudragit E PO)	100–130	---	8	---	Haake™ MiniLab II (Thermo Fisher)	150	---	---	100	0.4	0.2	Replicator 2 (MakerBot Ind.)	ER disks	[42]
	90–100	---	80	---	Haake™ MiniCTW (Thermo Fisher)	135	90	150	100	0.4	0.2	MakerWare (Makerbot Ind.)	IR tablets	[29]
	100, 120	100	---	1.75	Haake™ MiniCTW (Thermo Fisher)	230	---	---	---	---	---	Replicator 2 (MakerBot Ind.)	---	[44]
	90–100	---	0.8	---	Haake™ MiniCTW (Thermo Fisher)	135	90	150	100	0.4	0.2	MakerWare (Makerbot Ind.)	CR tablets	[74]
	130	---	0.6	1.50	Haake™ MiniCTW (Thermo Fisher)	140	90	150	100	---	0.2	Replicator 2 (Makerbot Ind.)	IR; ER capsules	[26]
Poly(ethyl acrylate-*co*-methyl methacrylate-*co*-trimethylammonio ethyl methacrylate chloride) 1:2:0.2 (Eudragit RL)	160	20	---	1.6–1.7	Pharmalab HME 16 (Thermo Fisher)	140–180	20–40	46.65–74.55	20–40	0.4	0.20	XXL Pro (Prodim Int., Helmond, The Netherlands)	SR tablets	[72]
	160	20	---	1.75	Pharmalab HME 16 (Thermo Fisher)	180	15	---	10, 15, 20, 25, 30, 40	0.4	---	XXL Pro (Prodim Int.)	SR tablets	[18]
	110	---	---	1.50	Noztec Pro Hot Melt (Noztec)	170	90	150	100	---	0.2	Replicator 2 (MakerBot Ind.)	CR tablets	[5]
	140	35	---	1.75	Filabot Original^®^ (Filabot)	170	70	90	100	---	0.20	Replicator 2 (MakerBot Ind.)	SR tablets	[59]
	130	---	0.6	1.50	---	170	90	150	100	---	0.20	Replicator 2 (MakerBot Ind.)	IR and ER tablets	[26]
	120	95	60	1.80	Haake™ MiniLab II (Thermo Fisher)	160	---	---	100	0.4	0.30	Replicator 2 (MakerBot Ind.)	CR disks	[25]
Poly(methacylic acid-*co*-methyl methacrylate) 1:1 (Eudragit L)	160	80	120	1.80	Haake™ MiniLab II (Thermo Fisher)	160	---	---	100	0.4	0.30	Replicator 2 (MakerBot Ind.)	CR disks	[25]
Poly(ethyl acrylate-*co*-methyl methacrylate-*co*-trimethylammonioethyl methacrylate chloride) 1:2:0.1 (Eudragit RS)	130	---	0.6	1.50	---	150	90	150	100	---	0.20	Replicator 2 (MakerBot Ind.)	IR and ER tablets	[26]
	55	0.94	---	3.00	---	155	144	40	100	0.5	0.10	Multirap M420 (Multec GmbH).	IR tablets	[69]
OTHER POLYMERS														
Polyvinyl caprolactam–polyvinyl acetate–polyethylene glycol graft copolymer (PCL: PVA: PEG)	120	80	80	1.80	Haake™ MiniLab II (Thermo Fisher)	200	---	---	100	0.4	0.30	Replicator 2 (MakerBot Ind.)	CR disks	[25]
	120	100	80	1.75	Haake™ MiniLab II (Thermo Fisher)	150	---	---	100	---	0.20	Replicator 2 (MakerBot Ind.)	CR disks	[42]
	100, 110, 120	100	---	1.75	Haake™ MiniCTW (Thermo Fisher)	230	---	---	---	---	---	Replicator 2 (MakerBot Ind.)	---	[44]
Polyvinyl alcohol/polyethylene glycol graft copolymer (PVA/PEG)	160	100	80	1.80	Haake™ MiniLab II (Thermo Fisher)	180 ---	---	---	100	0.4	0.30	Replicator 2 (MakerBot Ind.)	IR disks	[25]
	160	100	80	1.80	Haake™ MiniLab II (Thermo Fisher)	180 ---	---	---	100	0.3 0.4	0.10	Replicator 2 (MakerBot Ind.)	PR Capsular devices	[34]
	145	15	---	1.75	Noztec Pro Hot Melt (Noztec)	155 40	3	25	100	0.5	0.10	Replicator 2 (MakerBot Ind.)	CR tablets	[35]
	175	1	---	1.75	Noztek, (Noztek)	198–204	15	25	75, 70, 100	-	-	---	IR films	[90]
	150–170	200	---	1.55	(Thermo Fisher)	210 RT	45	150	60–100	0.4	0.10	Replicator 2 (MakerBot Ind.)	IR tablets	[61]
Polyethylene oxide (PEO)	60	---	---	1.60	Noztek Pro (Noztek)	165 RT	70	60	40	0.4	0.10	Wanhao Duplicator 4 (Wanhao)	IR films	[93]
	70	35	---	1.50	Haake™ MiniCTW (Thermo Fisher)	110 40	50	150	100	0.4	0.20	Replicator 2 (MakerBot Ind.)	IR tablets	[73]
	80	35	---	1.50	Haake™ MiniCTW (Thermo Fisher)	145 40	50	150	100	0.4	0.20	Replicator 2 (MakerBot Ind.)	IR tablets	
Poly(e-caprolactone)	65	---	---	2.00	Noztec Pro Hot Melt (Noztec)	95	90	150	100	---	0.2	Replicator 2 (MakerBot Ind.)	CR tablets	[5]
	100	30	---	1.75	Haake™ MiniCTW (Thermo Fisher)	100	45	150	10, 30	---	0.10	Replicator 2 (MakerBot Ind.)	CR implants	[94]
	47	0.94	---	3.00	---	53	144	40	100	0.5	0.10	Multirap M420 (Multec GmbH).	IR tablets	[69]
Ethylene vinyl acetate (EVA)	110	10	---	1.5, 2.5	Haake™ MiniCTW (Thermo Fisher)	165	10	150	100	---	0.1	Replicator 2 (MakerBot Ind.)	CR implants	[64]

--- Information not available; RT—room temperature; CR—controlled release; ER—extended release; IR—immediate release; MR—modified release; PR—pulsatile release; SR—sustained release.

## Abbreviations

3D	Three-dimensional
4-ASA	4-Aminosalicylic acid
5-ASA	5-Aminosalicylic acid
API	Active pharmaceutical ingredient
ASD	Amorphous solid dispersion
ATM	Atomic force microscopy
DMA	Dynamic mechanical analysis
DSC	Differential scanning calorimetry
EC	Ethyl cellulose
EVA	Ethylene vinyl acetate
FDM	Fused deposition modelling
FTIR	Fourier-Transform Infrared Spectroscopy
HEC	Hydroxyethyl cellulose
HME	Hot-melt extrusion
HPC	Hydroxypropyl cellulose
HPLC	High-pressure liquid chromatography
HPMC	Hydroxypropyl methylcellulose
HPMCAS	Hydroxypropyl methylcellulose acetate succinate
MC	Methyl cellulose
MDSC	Modulated differential scanning calorimetry
MW	Molecular weight
NIR	Near infrared spectroscopy
PEG	Poly(ethylene glycol)
PEO	Poly(ethylene oxide)
PCL	Polyvinyl caprolactam
PCLa	Polycaprolactone
PLA	Polylactic acid
PLM	Polarized light microscopy
PVA	Polyvinyl alcohol
PVAc	Polyvinyl acetate
PVP	Polyvinylpyrrolidone
SEM	Scanning electron microscopy
ssNMR	Solid state nuclear magnetic resonance
Tg	Glass transition temperature
Tm	Melting point
TBP	Tribasic phosphate sodium
TCP	Tri-calcium phosphate
TEC	Triethyl citrate
TEM	Transmission electron microscopy
TGA	Thermogravimetric analysis
XRPD	X-Ray powder diffraction

## References

[1] Alomari M., Mohamed F.H., Basit A.W., Gaisford S. (2015). Personalised dosing: Printing a dose of one’s own medicine. Int. J. Pharm..

[2] Genina N., Boetker J.P., Colombo S., Harmankaya N., Rantanen J., Bohr A. (2017). Anti-tuberculosis drug combination for controlled oral delivery using 3D printed compartmental dosage forms: From drug product design to in vivo testing. J. Control. Release.

[3] Goyanes A., Robles Martinez P., Buanz A., Basit A.W., Gaisford S. (2015). Effect of geometry on drug release from 3D printed tablets. Int. J. Pharm..

[4] Zhang J., Feng X., Patil H., Tiwari R.V., Repka M.A. (2017). Coupling 3D printing with hot-melt extrusion to produce controlled-release tablets. Int. J. Pharm..

[5] Beck R.C.R., Chaves P.S., Goyanes A., Vukosavljevic B., Buanz A., Windbergs M., Basit A.W., Gaisford S. (2017). 3D printed tablets loaded with polymeric nanocapsules: An innovative approach to produce customized drug delivery systems. Int. J. Pharm..

[6] Pardeike J., Strohmeier D.M., Schrödl N., Voura C., Gruber M., Khinast J.G., Zimmer A. (2011). Nanosuspensions as advanced printing ink for accurate dosing of poorly soluble drugs in personalized medicines. Int. J. Pharm..

[7] Vijayavenkataraman S., Fuh J.Y.H., Lu W.F. (2017). 3D printing and 3D bioprinting in pediatrics. Bioengineering.

[8] Khaled S.A., Burley J.C., Alexander M.R., Yang J., Roberts C.J. (2015). 3D printing of five-in-one dose combination polypill with defined immediate and sustained release profiles. J. Control. Release.

[9] Webster R., Castellano J.M., Onuma O.K. (2017). Putting polypills into practice: Challenges and lessons learned. Lancet.

[10] Huffman M.D., Xavier D., Perel P. (2017). Uses of polypills for cardiovascular disease and evidence to date. Lancet.

[11] Hsiao W.K., Lorber B., Reitsamer H., Khinast J. (2018). 3D printing of oral drugs: A new reality or hype?. Expert Opin. Drug Deliv..

[12] Fuenmayor E., Forde M., Healy A.V., Devine D.M., Lyons J.G., McConville C., Major I. (2019). Comparison of fused-filament fabrication to direct compression and injection molding in the manufacture of oral tablets. Int. J. Pharm..

[13] Chai X., Chai H., Wang X., Yang J., Li J., Zhao Y., Cai W., Tao T., Xiang X. (2017). Fused deposition modeling (FDM) 3D printed tablets for intragastric floating delivery of domperidone. Sci. Rep..

[14] Ursan I.D., Chiu L., Pierce A. (2013). Three-dimensional drug printing: A structured review. J. Am. Pharm. Assoc..

[15] Norman J., Madurawe R.D., Moore C.M.V., Khan M.A., Khairuzzaman A. (2017). A new chapter in pharmaceutical manufacturing: 3D-printed drug products. Adv. Drug Deliv. Rev..

[16] Goyanes A., Det-Amornrat U., Wang J., Basit A.W., Gaisford S. (2016). 3D scanning and 3D printing as innovative technologies for fabricating personalized topical drug delivery systems. J. Control. Release.

[17] Tagami T., Nagata N., Hayashi N., Ogawa E., Fukushige K., Sakai N., Ozeki T. (2018). Defined drug release from 3D-printed composite tablets consisting of drug-loaded polyvinylalcohol and a water-soluble or water-insoluble polymer filler. Int. J. Pharm..

[18] Korte C., Quodbach J. (2018). Formulation development and process analysis of drug-loaded filaments manufactured via hot-melt extrusion for 3D-printing of medicines. Pharm. Dev. Technol..

[19] Schubert C., Van Langeveld M.C., Donoso L.A. (2014). Innovations in 3D printing: A 3D overview from optics to organs. Br. J. Ophthalmol..

[20] Berman B. (2012). 3-D printing: The new industrial revolution. Bus. Horiz..

[21] Patil H., Tiwari R.V., Repka M.A. (2016). Hot-melt extrusion: From Theory to application in pharmaceutical formulation. AAPS Pharmscitech.

[22] Tan D.K., Maniruzzaman M., Nokhodchi A. (2018). Advanced pharmaceutical applications of hot-melt extrusion coupled with fused deposition modelling (FDM) 3D printing for personalised drug delivery. Pharmaceutics.

[23] Kushwaha S. (2018). Application of hot melt extrusion in pharmaceutical 3D printing. J. Bioequiv. Availab..

[24] Araújo M.R.P., Sa-Barreto L.L., Gratieri T., Gelfuso G.M., Cunha-Filho M. (2019). The digital pharmacies era: How 3D printing technology using fused deposition modeling can become a reality. Pharmaceutics.

[25] Melocchi A., Parietti F., Maroni A., Foppoli A., Gazzaniga A., Zema L. (2016). Hot-melt extruded filaments based on pharmaceutical grade polymers for 3D printing by fused deposition modeling. Int. J. Pharm..

[26] Pietrzak K., Isreb A., Alhnan M.A. (2015). A flexible-dose dispenser for immediate and extended release 3D printed tablets. Eur. J. Pharm. Biopharm..

[27] Goyanes A., Buanz A.B.M., Basit A.W., Gaisford S. (2014). Fused-filament 3D printing (3DP) for fabrication of tablets. Int. J. Pharm..

[28] Verstraete G., Samaro A., Grymonpré W., Vanhoorne V., Snick B.V., Boone M.N., Hellemans T., Hoorebeke L.V., Remon J.P., Vervaet C. (2018). 3D printing of high drug loaded dosage forms using thermoplastic polyurethanes. Int. J. Pharm..

[29] Sadia M., Sośnicka A., Arafat B., Isreb A., Ahmed W., Kelarakis A., Alhnan M.A. (2016). Adaptation of pharmaceutical excipients to FDM 3D printing for the fabrication of patient-tailored immediate release tablets. Int. J. Pharm..

[30] Dizon J.R.C., Espera A.H., Chen Q., Advincula R.C. (2018). Mechanical characterization of 3D-printed polymers. Addit. Manuf..

[31] Smith D.M., Kapoor Y., Klinzing G.R., Procopio A.T. (2018). Pharmaceutical 3D printing: Design and qualification of a single step print and fill capsule. Int. J. Pharm..

[32] Long J., Gholizadeh H., Lu J., Seyfoddin A. (2017). Review: Application of fused deposition modelling (FDM) method of 3D printing in drug delivery. Curr. Pharm. Des..

[33] Sandler N., Preis M. (2016). Printed drug-delivery systems for improved patient treatment. Trends Pharmacol. Sci..

[34] Maroni A., Melocchi A., Parietti F., Foppoli A., Zema L., Gazzaniga A. (2017). 3D printed multi-compartment capsular devices for two-pulse oral drug delivery. J. Control. Release.

[35] Goyanes A., Fernández-Ferreiro A., Majeed A., Gomez-Lado N., Awad A., Luaces-Rodríguez A., Gaisford S., Aguiar P., Basit A.W. (2018). PET/CT imaging of 3D printed devices in the gastrointestinal tract of rodents. Int. J. Pharm..

[36] Arafat B., Wojsz M., Isreb A., Forbes R.T., Isreb M., Ahmed W., Arafat T., Alhnan M.A. (2018). Tablet fragmentation without a disintegrant: A novel design approach for accelerating disintegration and drug release from 3D printed cellulosic tablets. Eur. J. Pharm. Sci..

[37] Bruce L.D., Shah N.H., Waseem Malick A., Infeld M.H., McGinity J.W. (2005). Properties of hot-melt extruded tablet formulations for the colonic delivery of 5-aminosalicylic acid. Eur. J. Pharm. Biopharm..

[38] Skowyra J., Pietrzak K., Alhnan M.A. (2015). Fabrication of extended-release patient-tailored prednisolone tablets via fused deposition modelling (FDM) 3D printing. Eur. J. Pharm. Sci..

[39] Warsi M.H., Yusuf M., Al Robaian M., Khan M., Alsaab H., Muheem A., Khan S. (2018). 3D printing methods for pharmaceutical manufacturing: Opportunity and challenges. Curr. Pharm. Des..

[40] Sadia M., Arafat B., Ahmed W., Forbes R.T., Alhnan M.A. (2018). Channelled tablets: An innovative approach to accelerating drug release from 3D printed tablets. J. Control. Release.

[41] Fuenmayor E., Forde M., Healy A.V., Devine D.M., Lyons J.G., McConville C., Major I. (2018). Material considerations for fused-filament fabrication of solid dosage forms. Pharmaceutics.

[42] Alhijjaj M., Belton P., Qi S. (2016). An investigation into the use of polymer blends to improve the printability of and regulate drug release from pharmaceutical solid dispersions prepared via fused deposition modeling (FDM) 3D printing. Eur. J. Pharm. Biopharm..

[43] Saviano M., Aquino R.P., Del Gaudio P., Sansone F., Russo P. (2019). Poly(vinyl alcohol) 3D printed tablets: The effect of polymer particle size on drug loading and process efficiency. Int. J. Pharm..

[44] Nasereddin J.M., Wellner N., Alhijjaj M., Belton P., Qi S. (2018). Development of a simple mechanical screening method for predicting the feedability of a pharmaceutical FDM 3D printing filament. Pharm. Res..

[45] Goyanes A., Buanz A.B., Hatton G.B., Gaisford S., Basit A.W. (2004). 3D printing of modified-release aminosalicylate (4-ASA and 5-ASA) tablets. Eur. J. Pharm. Biopharm..

[46] Lim S.H., Kathuria H., Tan J.J.Y., Kang L. (2018). 3D printed drug delivery and testing systems—A passing fad or the future?. Adv. Drug Deliv. Rev..

[47] Kollamaram G., Croker D.M., Walker G.M., Goyanes A., Basit A.W., Gaisford S. (2018). Low temperature fused deposition modeling (FDM) 3D printing of thermolabile drugs. Int. J. Pharm..

[48] Zhang A.J., Yang W., Vo A.Q., Feng X. (2017). Hydroxypropyl methylcellulose-based controlled release dosage by melt extrusion and 3D printing: Structure and drug release correlation. Carbohydr. Polym..

[49] Goyanes A., Fina F., Martorana A., Sedough D., Gaisford S., Basit A.W. (2017). Development of modified release 3D printed tablets (printlets) with pharmaceutical excipients using additive manufacturing. Int. J. Pharm..

[50] Goyanes A., Wang J., Buanz A., Martínez-Pacheco R., Telford R., Gaisford S., Basit A.W. (2015). 3D printing of medicines: Engineering novel oral devices with unique design and drug release characteristics. Mol. Pharm..

[51] Pereira B.C., Isreb A., Forbes R.T., Dores F., Habashy R. (2019). ‘Temporary Plasticiser’: A novel solution to fabricate 3D printed patient- centred cardiovascular ‘Polypill’ architectures. Eur. J. Pharm. Biopharm..

[52] Charoenying T., Patrojanasophon P., Ngawhirunpat T., Rojanarata T., Akkaramongkolporn P., Opanasopit P. (2020). Fabrication of floating capsule-in- 3D-printed devices as gastro-retentive delivery systems of amoxicillin. J. Drug Deliv. Sci. Technol..

[53] Jamróz W., Kurek M., Szafraniec-Szczęsny J., Czech A., Gawlak K., Knapik-Kowalczuk J., Leszczyński B., Wróbel A., Paluch M., Jachowicz R. (2020). Speed it up, slow it down…An issue of bicalutamide release from 3D printed tablets. Eur. J. Pharm. Sci..

[54] Matijašić G., Gretić M., Vinčić J., Poropat A., Cuculić L., Rahelić T. (2019). Design and 3D printing of multi-compartmental PVA capsules for drug delivery. J. Drug Deliv. Sci. Technol..

[55] Palekar S., Nukala P.K., Mishra S.M., Kipping T., Patel K. (2019). Application of 3D printing technology and quality by design approach for development of age-appro- priate pediatric formulation of baclofen. Int. J. Pharm..

[56] Okwuosa T.C., Pereira B.C., Arafat B., Cieszynska M., Isreb A., Alhnan M.A. (2017). Fabricating a shell-core delayed release tablet using dual FDM 3D printing for patient-centred therapy. Pharm. Res..

[57] Ilyés K., Balogh A., Casian T., Igricz T., Borbás E., Démuth B., Vass P., Menyhárt L., Kovács N.K., Marosi G. (2019). 3D floating tablets: Appropriate 3D design from the perspective of different in vitro dissolution testing methodologies. Int. J. Pharm..

[58] Okwuosa T.C., Stefaniak D., Arafat B., Isreb A., Wan K.W., Alhnan M.A. (2016). A Lower temperature FDM 3D printing for the manufacture of patient-specific immediate release tablets. Pharm. Res..

[59] Gioumouxouzis C.I., Baklavaridis A., Katsamenis O.L., Markopoulou C.K., Bouropoulos N., Tzetzis D., Fatouros D.G. (2018). A 3D printed bilayer oral solid dosage form combining metformin for prolonged and glimepiride for immediate drug delivery. Eur. J. Pharm. Sci..

[60] Li Q., Wen H., Jia D., Guan X., Pan H., Yang Y., Yu S., Zhu Z., Xiang R., Pan W. (2017). Preparation and investigation of controlled-release glipizide novel oral device with three-dimensional printing. Int. J. Pharm..

[61] Solanki N.G., Tahsin M., Shah A.V., Serajuddin A.T.M. (2018). Formulation of 3D printed tablet for rapid drug release by fused deposition modeling: Screening polymers for drug release, drug-polymer miscibility and printability. J. Pharm. Sci..

[62] Wei C., Solanki N.G., Vasoya J.M., Shah A.V., Serajuddin A.T.M. (2020). Development of 3D printed tablets by fused deposition modeling using polyvinyl alcohol as a polymeric matrix for rapid drug release. J. Pharm. Sci..

[63] Yang Y., Wang H., Li H., Ou Z., Yang G. (2018). 3D printed tablets with internal scaffold structure using ethyl cellulose to achieve sustained ibuprofen release. Eur. J. Pharm. Sci..

[64] Genina N., Holländer J., Jukarainen H., Mäkilä E., Salonen J., Sandler N. (2016). Ethylene vinyl acetate (EVA) as a new drug carrier for 3D printed medical drug delivery devices. Eur. J. Pharm. Sci..

[65] Scoutaris N., Ross S.A., Douroumis D. (2018). 3D printed “starmix” drug loaded dosage forms for paediatric applications. Pharm. Res..

[66] Kimura S.I., Ishikawa T., Iwao Y., Itai S., Kondo H. (2019). Fabrication of zero-order sustained-release floating tablets via fused depositing modeling 3D printer. Chem. Pharm. Bull..

[67] Boetker J., Water J.J., Aho J., Arnfast L., Bohr A., Rantanen J. (2016). Modifying release characteristics from 3D printed drug-eluting products. Eur. J. Pharm. Sci..

[68] Kempin W., Domsta V., Grathoff G., Brecht I., Semmling B., Tillmann S., Weitschies W., Seidlitz A. (2018). Immediate release 3D-printed tablets produced via fused deposition modeling of a thermo-sensitive drug. Pharm. Res..

[69] Kempin W., Franz C., Koster L.C., Schneider F., Bogdahn M., Weitschies W., Seidlitz A. (2017). Assessment of different polymers and drug loads for fused deposition modeling of drug loaded implants. Eur. J. Pharm. Biopharm..

[70] Van Nguyen H., Nguyen V.H., Lee B.-J. (2016). Dual release and molecular mechanism of bilayered aceclofenac tablet using polymer mixture. Int. J. Pharm..

[71] Dumpa N.R., Bandari S., Repka M.A. (2020). Novel gastroretentive floating pulsatile drug delivery system produced via hot-melt extrusion and fused deposition modeling 3D printing. Pharmaceutics.

[72] Korte C., Quodbach J. (2018). 3D-printed network structures as controlled-release drug delivery systems: Dose adjustment, API release analysis and prediction. AAPS Pharmscitech.

[73] Isreb A., Baj K., Wojsz M., Isreb M., Peak M., Alhnan M.A. (2019). 3D printed oral theophylline doses with innovative ‘radiator-like’ design: Impact of polyethylene oxide (PEO) molecular weight. Int. J. Pharm..

[74] Arafat B., Qinna N., Cieszynska M., Forbes R.T., Alhnan M.A. (2018). Tailored on demand anti-coagulant dosing: An in vitro and in vivo evaluation of 3D printed purpose-designed oral dosage forms. Eur. J. Pharm. Biopharm..

[75] Sarabu S., Bandari S., Kallakunta V.R., Tiwari R., Patil H., Repka M.A. (2019). An update on the contribution of hot-melt extrusion technology to novel drug delivery in the twenty-first century: Part II. Expert Opin. Drug Deliv..

[76] Flory P. (1953). Principles of Polymer Chemistry.

[77] Thakkar R., Thakkar R., Pillai A., Ashour E.A., Repka M.A. (2020). Systematic screening of pharmaceutical polymers for hot melt extrusion processing: A comprehensive review. Int. J. Pharm..

[78] Balani K., Verma V., Agarwal A., Narayan R. (2015). Physical, thermal, and mechanical properties of polymers. Biosurfaces.

[79] Kolter K., Karl M., Gryczke A. (2012). Hot-Melt Extrusion with BASF Pharma Polymers: Extrusion Compendium.

[80] Prasad L.K., Smyth H. (2016). 3D Printing technologies for drug delivery: A review. Drug Dev. Ind. Pharm..

[81] Shahriar B.B., France C., Valerie N., Arthur C., Christian G. (2017). Toward improvement of the properties of parts manufactured by FFF (fused filament fabrication) through understanding the influence of temperature and rheological behaviour on the coalescence phenomenon. AIP Conference Procceedings.

[82] Mehuys E., Remon J.P., Vervaet C. (2005). Production of enteric capsules by means of hot-melt extrusion. Eur. J. Pharm. Sci..

[83] Melocchi A., Parietti F., Loreti G., Maroni A., Gazzaniga A., Zema L. (2015). 3D printing by fused deposition modeling (FDM) of a swellable/erodible capsular device for oral pulsatile release of drugs. J. Drug Deliv. Sci. Technol..

[84] Khaled S.A., Burley J.C., Alexander M.R., Roberts C.J. (2014). Desktop 3D printing of controlled release pharmaceutical bilayer tablets. Int. J. Pharm..

[85] Iftimi L.D., Edinger M., Bar-Shalom D., Rantanen J., Genina N. (2019). Edible solid foams as porous substrates for inkjet-printable pharmaceuticals. Eur. J. Pharm. Biopharm..

[86] Zhang J., Xu P., Vo A.Q., Bandari S., Yang F., Durig T., Repka M.A. (2019). Development and evaluation of pharmaceutical 3D printability for hot melt extruded cellulose-based filaments. J. Drug Deliv. Sci. Technol..

[87] Melocchi A., Uboldi M., Maroni A., Foppoli A., Palugan L., Zema L., Gazzaniga A. (2020). 3D printing by fused deposition modeling of single- and multi-compartment hollow systems for oral delivery–A review. Int. J. Pharm..

[88] Cameron G.G., Ingram M.D., Qureshi M.Y., Gearing H.M., Costa L., Camino G., Giuria V.P. (1989). The thermal degradation of poly(Ethylene Oxide) and its complex with NaCNS. Eur. Polym. J..

[89] Goyanes A., Chang H., Sedough D., Hatton G.B., Wang J., Buanz A., Gaisford S., Basit A.W. (2015). Fabrication of controlled-release budesonide tablets via desktop (FDM) 3D printing. Int. J. Pharm..

[90] Jamróz W., Kurek M., Łyszczarz E., Szafraniec J., Knapik-Kowalczuk J., Syrek K., Paluch M., Jachowicz R. (2017). 3D printed orodispersible films with Aripiprazole. Int. J. Pharm..

[91] Goyanes A., Scarpa M., Kamlow M., Gaisford S., Basit A.W., Orlu M. (2017). Patient acceptability of 3D printed medicines. Int. J. Pharm..

[92] Holländer J., Hakala R., Suominen J., Moritz N., Yliruusi J., Sandler N. (2018). 3D printed UV light cured polydimethylsiloxane devices for drug delivery. Int. J. Pharm..

[93] Ehtezazi T., Algellay M., Islam Y., Roberts M., Dempster N.M., Sarker S.D. (2018). The Application of 3D printing in the formulation of multilayered fast dissolving oral films. J. Pharm. Sci..

[94] Holländer J., Genina N., Jukarainen H., Khajeheian M., Rosling A., Mäkilä E., Sandler N. (2016). Three-dimensional printed PCL-based implantable prototypes of medical devices for controlled drug delivery. J. Pharm. Sci..

[95] United States Pharmacopeial Convention Inc. (2019). United States Pharmacopeia.

[96] Council of Europe (2021). European Pharmacopeia.

[97] Tagami T., Fukushige K., Ogawa E., Hayashi N., Ozeki T. (2017). 3D printing factors important for the fabrication of polyvinylalcohol filament-based tablets. Biol. Pharm. Bull..

[98] Awasthi R., Manchanda S., Das P., Velu V., Malipeddi H., Pabreja K., Pinto T.D.J.A., Gupta G., Dua K., Parambath A. (2018). *Poly(vinylpyrrolidone)*. Engineering of Biomaterials for Drug Delivery Systems.

[99] Kollidon VA 64 Fine Pharmaceuticals. https://pharmaceutical.basf.com/global/en/drug-formulation/products/kollidon-va64-fine.html.

[100] BASF (2013). Soluble Kollidon® Grades.

[101] Chavan R.B., Rathi S., Jyothi V.G.S.S., Shastri N.R. (2019). Cellulose based polymers in development of amorphous solid dispersions. Asian J. Pharm. Sci..

[102] Edgar K.J., Buchanan C.M., Debenham J.S., Rundquist P.A., Seiler B.D., Shelton M.C., Tindall D. (2001). Advances in cellulose ester performance and application. Prog. Polym. Sci..

[103] Gómez-Carracedo A., Alvarez-Lorenzo C., Gómez-Amoza J.L., Concheiro A. (2003). Chemical structure and glass transition temperature of non-ionic cellulose ethers. J. Therm. Anal. Calorim..

[104] Dürig T., Karan K. (2019). Binders in Wet Granulation. Handbook of Pharmaceutical Wet Granulation.

[105] Picker-Freyer K.M., Dürig T. (2007). Physical mechanical and tablet formation properties of hydroxypropylcellulose: In pure form and in mixtures. AAPS Pharmscitech.

[106] Siepmann J., Peppas N.A. (2012). Modeling of drug release from delivery systems based on hydroxypropyl methylcellulose (HPMC). Adv. Drug Deliv. Rev..

[107] Karkri M. (2017). Thermal Conductivity of Biocomposite Materials. Biopolymer Composites in Electronics.

[108] Gupta S.S., Solanki N., Serajuddin A.T.M. (2016). Investigation of Thermal and Viscoelastic Properties of Polymers Relevant to Hot Melt Extrusion, IV: Affinisol^TM^ HPMC HME Polymers. AAPS Pharmscitech.

[109] Brady J., Dürig T., Lee P.I., Li J.-X., Qiu Y., Chen Y., Zhang G.G.Z., Yu L., Mantri R.V. (2017). Polymer Properties and Characterization. Developing Solid Oral Dosage Forms.

[110] Zema L., Loreti G., Melocchi A., Maroni A., Palugan L., Gazzaniga A. (2013). Gastroresistant capsular device prepared by injection molding. Int. J. Pharm..

[111] Dong Z., Choi D.S. (2008). Hydroxypropyl Methylcellulose Acetate Succinate: Potential Drug—Excipient Incompatibility. AAPS Pharmscitech.

[112] Tanno F., Nishiyama Y., Kokubo H., Obara S. (2004). Evaluation of Hypromellose Acetate Succinate (HPMCAS) as a Carrier in Solid Dispersions. Drug Dev. Ind. Pharm..

[113] Joshi G.V., Kevadiya B.D., Bajaj H.C. (2010). Controlled release formulation of ranitidine-containing montmorillonite and Eudragit^®^ E-100. Drug Dev. Ind. Pharm..

[114] Nguyen C.A., Konan-Kouakou Y.N., Allémann E., Doelker E., Quintanar-Guerrero D., Fessi H., Gurny R. (2006). Preparation of surfactant-free nanoparticles of methacrylic acid copolymers used for film coating. AAPS Pharmscitech.

[115] Kerdsakundee N., Mahattanadul S., Wiwattanapatapee R. (2015). Development and evaluation of gastroretentive raft forming systems incorporating curcumin-Eudragit^®^ EPO solid dispersions for gastric ulcer treatment. Eur. J. Pharm. Biopharm..

[116] Nagy Z.K., Balogh A., Azs B.A.L., Farkas A., Marosi G.Y.O. (2012). Comparison of Electrospun and Extruded Soluplus R -Based Solid. J. Pharm. Sci..

[117] Mahapatro A., Singh D.K. (2011). Biodegradable nanoparticles are excellent vehicle for site directed in-vivo delivery of drugs and vaccines. J. Nanobiotechnol..

[118] Kiefer D., Yu L., Fransson E., Gómez A., Primetzhofer D., Amassian A., Campoy-Quiles M., Müller C. (2017). A Solution-Doped Polymer Semiconductor:Insulator Blend for Thermoelectrics. Adv. Sci..

[119] Oliveira M., Cardoso A., Viana M., Lins V. (2018). The causes and effects of degradation of encapsulant ethylene vinyl acetate copolymer (EVA) in crystalline silicon photovoltaic modules: A review. Renew. Sustain. Energy Rev..

[120] Solanki N.G., Kathawala M., Serajuddin A.T.M. (2019). Effects of Surfactants on Itraconazole-Hydroxypropyl Methylcellulose Acetate Succinate Solid Dispersion Prepared by Hot Melt Extrusion III: Tableting of Extrudates and Drug Release From Tablets. J. Pharm. Sci..

[121] Ilyés K., Kovács N.K., Balogh A., Borbás E., Farkas B., Casian T., Marosi G., Tomuță I., Nagy Z.K. (2019). The applicability of pharmaceutical polymeric blends for the fused deposition modelling (FDM) 3D technique: Material considerations–printability–process modulation, with consecutive effects on in vitro release, stability and degradation. Eur. J. Pharm. Sci..

[122] Saerens L., Dierickx L., Lenain B., Vervaet C., Remon J.P., Beer D.T. (2011). Raman spectroscopy for the in-line polymer-drug quantification and solid state characterization during a pharmaceutical hot-melt extrusion process. Eur. J. Pharm. Biopharm..

[123] Aho J., Boetker J.P., Baldursdottir S., Rantanen J. (2015). Rheology as a tool for evaluation of melt processability of innovative dosage forms. Int. J. Pharm..

[124] Costa S.F., Duarte F.M., Covas J.A. (2017). Estimation of filament temperature and adhesion development in fused deposition techniques. J. Mater. Process. Technol..

[125] Sun Q., Rizvi G.M., Bellehumeur C.T., Gu P. (2008). Effect of processing conditions on the bonding quality of FDM polymer filaments. Rapid Prototyp. J..

[126] Konta A., García-Piña M., Serrano D. (2017). Personalised 3D Printed Medicines: Which Techniques and Polymers Are More Successful?. Bioengineering.

[127] Goole J., Amighi K. (2016). 3D printing in pharmaceutics: A new tool for designing customized drug delivery systems. Int. J. Pharm..

[128] Lewis J.A., Gratson G.M. (2004). Direct writing in three dimensions. Mater. Today.

[129] Elbadawi M. (2019). Rheological and Mechanical Investigation into the Effect of Different Molecular Weight Poly(ethylene glycol)s on Polycaprolactone-Ciprofloxacin Filaments. ACS Omega.

[130] Tanner R.I., Keentok M. (1983). Shear Fracture in Cone-Plate Rheometry. J. Rheol..

[131] Azad M.A., Olawuni D., Kimbell G., Badruddoza A.Z.M., Hossain M.S., Sultana T. (2020). Polymers for extrusion-based 3D printing of pharmaceuticals: A holistic materials–process perspective. Pharmaceutics.

[132] Kim M.H., Lee Y.W., Jung W.K., Oh J., Nam S.Y. (2019). Enhanced rheological behaviors of alginate hydrogels with carrageenan for extrusion-based bioprinting. J. Mech. Behav. Biomed. Mater..

[133] Shrivastava A. (2018). Plastic Properties and Testing. Introduction to Plastics Engineering.

[134] Venkataraman N., Rangarajan S., Matthewson M.J., Harper B., Safari A., Danforth S.C., Wu G., Langrana N., Guceri S., Yardimci A. (2000). Feedstock material property—Process relationships in fused deposition of ceramics (FDC). Rapid Prototyp. J..

[135] Ma X., Williams R.O. (2019). Characterization of amorphous solid dispersions: An update. J. Drug Deliv. Sci. Technol..

[136] Bellehumeur C., Li L., Sun Q., Gu P. (2004). Modeling of bond formation between polymer filaments in the fused deposition modeling process. J. Manuf. Process..

[137] Rösler J., Harders H., Bäker M. (2007). Mechanical behaviour of polymers. Mechanical Behaviour of Engineering Materials.

[138] Ebnesajjad S. (2014). Surface and Material Characterization Techniques. Surface Treatment of Materials for Adhesion Bonding.

[139] Vo C.L.N., Park C., Lee B.J. (2013). Current trends and future perspectives of solid dispersions containing poorly water-soluble drugs. Eur. J. Pharm. Biopharm..

[140] Messimer S.L., Patterson A.E., Muna N., Deshpande A.P., Rocha Pereira T. (2018). Characterization and Processing Behavior of Heated Aluminum-Polycarbonate Composite Build Plates for the FDM Additive Manufacturing Process. J. Manuf. Mater. Process..

[141] Heidemann H.M., Dotto M.E.R., Laurindo J.B., Carciofi B.A.M., Costa C. (2019). Cold plasma treatment to improve the adhesion of cassava starch films onto PCL and PLA surface. Colloids Surf. A Physicochem. Eng. Asp..

[142] Azman Mohammad Taib M.N., Julkapli N.M., Jawaid M., Thariq M., Saba N. (2019). 4—Dimensional stability of natural fiber-based and hybrid composites. Woodhead Publishing Series in Composites Science and Engineering.

[143] Vakili H., Kolakovic R., Genina N., Marmion M., Salo H., Ihalainen P., Peltonen J., Sandler N. (2015). Hyperspectral imaging in quality control of inkjet printed personalised dosage forms. Int. J. Pharm..

[144] Fina F., Goyanes A., Gaisford S., Basit A.W. (2017). Selective laser sintering (SLS) 3D printing of medicines. Int. J. Pharm..

[145] Boparai K.S., Singh R. (2018). Development of rapid tooling using fused deposition modeling. Addit. Manuf. Emerg. Mater..

[146] Goyanes A., Kobayashi M., Martínez-Pacheco R., Gaisford S., Basit A.W. (2016). Fused-filament 3D printing of drug products: Microstructure analysis and drug release characteristics of PVA-based caplets. Int. J. Pharm..

